# A wave-confining metasphere beamforming acoustic sensor for superior human-machine voice interaction

**DOI:** 10.1126/sciadv.adc9230

**Published:** 2022-09-28

**Authors:** Kejing Ma, Huyue Chen, Zhiyuan Wu, Xiangling Hao, Ge Yan, Wenbo Li, Lei Shao, Guang Meng, Wenming Zhang

**Affiliations:** ^1^ State Key Laboratory of Mechanical Systems and Vibration, School of Mechanical Engineering, Shanghai Jiao Tong University, Shanghai, 200240, P. R. China.; ^2^ University of Michigan–Shanghai Jiao Tong University Joint Institute, Shanghai Jiao Tong University, Shanghai, 200240, P. R. China.; ^3^ Interdisciplinary Research Center, Shanghai Jiao Tong University, Shanghai, 200240, P. R. China.

## Abstract

Highly sensitive, source-tracking acoustic sensing is essential for effective and natural human-machine interaction based on voice. It is a known challenge to omnidirectionally track sound sources under a hypersensitive rate with low noise interference using a compact sensor. Here, we present a unibody acoustic metamaterial spherical shell with equidistant defected piezoelectric cavities, referred to as the metasphere beamforming acoustic sensor (MBAS). It demonstrates a wave-confining capability and low self-noise, simultaneously achieving an outstanding intrinsic signal-to-noise ratio (72 dB) and an ultrahigh sensitivity (137 mV_pp_/Pa or −26.3 dBV), with a range spanning the daily phonetic frequencies (0 to 1500 Hz) and omnidirectional beamforming for the perception and spatial filtering of sound sources. Moreover, the MBAS-based auditory system is shown for high-performance audio cloning, source localization, and speech recognition in a noisy environment without any signal enhancement, revealing its promising applications in various voice interaction systems.

## Teaser

An acoustic metasphere reforms currently popular omnidirectional microphone arrays for superior voice-based interaction.

## INTRODUCTION

Intelligent machines and robots equipped with effective and natural human-machine interaction (HMI) technologies are critical for social communication, corporation, and exploration (*1*). Same as conversations, which are the most common and effortless way to communicate with people (*2*), voice-based HMI with robust sound sensing, speech recognition, and emotion perception has also been expected as one direction for straightforward interactions with machines. This requires acoustic sensors with combined ultrahigh signal-to-noise ratio (SNR) and sensitivity to discern desired sound waves in noisy surroundings and three-dimensional (3D) beamforming capability to locate and filtrate sound sources.

Currently, most commercial and similar-principled new acoustic sensors still rely on electret or microelectromechanical systems (MEMS) microphones (capacitive, piezoresistive, etc.) and their arrays, which are typically associated with a low sensitivity, high power consumption, or cumbersome signal processing systems. Therefore, it is usually an unsatisfactory experience to interact with present auditory systems, such as smart speakers and conference room microphone arrays. At the same time, high-sensitivity wearable and skin-attachable acoustic sensors with flexible materials have attracted a lot of research attention for their biointerfacing with the human body (*3*–*6*), and there are also works achieving broadband low-frequency flexible sensors to cover the frequency range of human voices. For example, flexible triboelectric auditory sensors with high sensitivity were fabricated and their potential in HMI systems was demonstrated (*7*–*9*). Multiband piezoelectric acoustic sensors were developed to broaden the resonant frequency to cover the phonetic frequencies (*10*–*12*). Nevertheless, these representative thin-film sensors do not show high SNR and sensitivity simultaneously, and they transduce sound waves efficiently only for the local pressure at their specific position without confining or amplifying the spatially low-density sound energy (*13*, *14*). They are also still required to form a larger sensor array in an interference-dominant, noisy environment to localize and extract desired source signals by spatial beamforming, which has been deployed in many sound-detecting applications (*15*–*17*).

Recently, various acoustic metamaterials have been introduced with unlimited possibilities for sound manipulation and control (*18*, *19*), showing their unique advantages in developing acoustic sensors for future voice-based HMI systems. Specifically, there are two main different types of designs of acoustic metamaterials, one is for passively amplifying weak sound wavefields while the other is for separation and localization of different overlapping sources. On the one hand, amplified sound detection is achieved by a number of works, including vibration confining based on defect planar metamaterial structures (*20*–*22*), wave compression based on strong anisotropic structures (*23*), and nonradiative sound transfer based on pairs of self-resonating subwavelength Mie metacavities (*24*). They represent an enhanced acoustic sensing strategy by pressure amplification using different passive metamaterial structures. On the other hand, a single regular sensor inside spatially designed acoustic metamaterials has been shown for the separation and localization of sound waves (*25*, *26*) and elastic vibrations (*27*) without using arrayed acoustic sensors. In these systems, either a metamaterial enclosure or a resonant coupling network encodes the response of the sensor in a direction-dependent manner, followed by a demodulation algorithm to reconstruct the source locations. However, it is still a challenge to dynamically and simultaneously track sound sources with different or similar incident angles in a noisy background, which has not been shown in all previous studies on acoustic metamaterials, let alone achieving accurate speech recognition in real time under such stringent conditions. This will require a functional combination of low-noise sound pressure amplification with fast source localization capabilities, leading to high-quality acoustic sensing and tracking in future voice-based HMI systems (*28*).

Here, we report an acoustic metamaterial–based spherical shell with an evenly distributed defect cavity array across the whole surface for sound pressure and vibrational energy confinement, where piezoelectric acoustic sensing is integrated inside each cavity. This unibody spherical shell structure, referred to as the metasphere beamforming acoustic sensor (MBAS) below, strongly confines sound pressure inside each cavity, which forms an omnidirectional beamforming array for source localization and directional signal reception. Without any signal processing, it achieves an outstanding SNR (72 dB) and an ultrahigh sensitivity (137 mV_pp_/Pa or −26.3 dBV) simultaneously near the international standard pitch (fig. S1) compared to most other existing sensors (fig. S2). At the same time, the MBAS shows high linearity, suitable broadband phonetic frequencies, and omnidirectional acoustic beamforming capability. We then demonstrate MBAS-based multitask HMI without signal amplification electronics, including audio reproduction, and real-time speech recognition and tracking for simultaneous sounds with similar incident angles in a conference room and a noisy factory. This work exhibits a prospective application of the MBAS for superior voice-based HMI systems in future intelligent machines.

## RESULTS

### Sensor design and principle

**Fig. 1.**
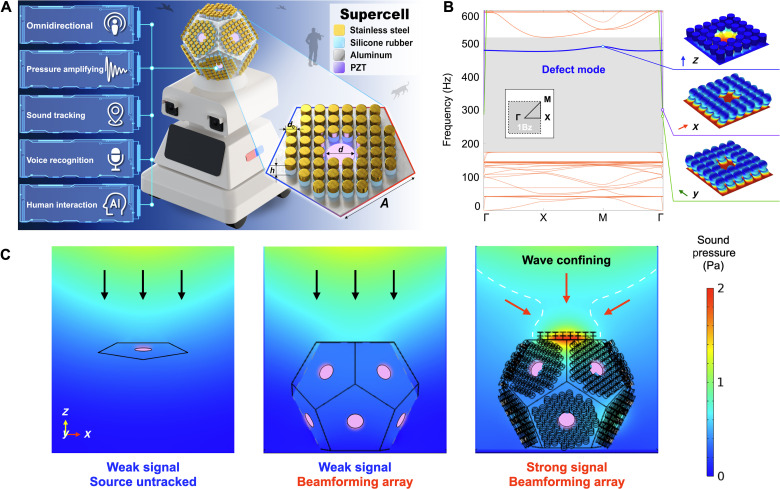
Overall concept and mechanism of the MBAS. (**A**) Schematic illustration of the design of the MBAS with a zoom-in of its pentagon-shaped supercell, together with its promising applications as a voice-based HMI for an intelligent service robot. (**B**) The acoustic frequency–wave number dispersive relation of one supercell, showing a pseudo-bandgap (gray shading area) embedding a defect vibrational mode (in blue), with the inset showing the first Brillouin zone for a planar metamaterial. Vibrational mode profiles are shown for the defect mode (out-of-plane vibration, *z* directional) and the two fast in-plane modes (*x* or *y* directional) inside the pseudo-bandgap. (**C**) Comparison of the simulated sound pressure distribution for three sensors, including a thin plate carrying a lead-zirconate-titanate (PZT) acoustic sensor, a spherical arrayed PZT sensor, and the MBAS, under 1-Pa plane-wave sound incident from the top.

Planar local resonance acoustic metamaterials are known for their acoustic bandgaps occurring at wavelengths an order of magnitude larger than their periodicity and, thus, can control low-frequency human phonetic sounds. In this work, we propose an acoustic metamaterial spherical shell consisting of multiple planar acoustic metamaterial plates, with evenly distributed defect cavities at the center of each plate for omnidirectional beamforming, referred to as the MBAS. This MBAS can function as the core of audio reproduction and speech recognition systems for intelligent voice interaction systems. The concept scheme and structural design of the MBAS, shown in Fig. 1A (detailed design with fabrication process in figs. S3 to S6), are mainly a 3D spherical structure approximated by a regular dodecahedron. Each face is a planar acoustic metamaterial thin plate, carrying a piezoelectric patch in the center defect for vibrational energy confining and transduction. The metamaterial is obtained by periodically attaching an array of resonators, short columns of stainless steel on silicone rubber, on a thin aluminum plate. A defect cavity is then formed by removing four resonators in the center of the plate, where the sound pressure is focused and the vibrational mode of interest is confined.

Here, we first obtain the theoretical signal output with respect to MBAS vibration by modeling the column resonators as spring-mass systems and the thin aluminum plate as Kirchhoff’s thin plate (as shown in fig. S7). On the basis of the Kirchhoff thin plate theory (*29*, *30*), the motion equation of locally resonant acoustic metamaterial plate is

$$D\nabla^{4}W(r)-\rho h\omega^{2}W(r)=\sum_{j=1}^{N}\sum_{R}f_{j}(r_{j}+R)\delta [r-(r_{j}+R)]$$ (1)

$$f(r_{j}+R)=K_{j}(u_{j}(r_{j}+R)-W(r_{j}+R))=\omega^{2}m_{j}u_{j}(r_{j}+R)$$ (2)

where *D* = *Eh*^3^/12(1 − *v*^2^) is the bending stiffness of the plate, in which *E*, ν, ρ, and *h* are the Young’s modulus, Poisson ratio, density, and thickness of the plate, respectively; *f_j_* is the force of the resonator on the plate at the position of *r_j_* + *R*, where *r_j_* = (*x_j_*, *y_j_*) is the coordinate of the *j*th resonator in the supercell; *R* = *ma*_1_ + *na*_2_, where *m* and *n* are integers; |*a*_1_| = |*a*_2_| = *a* is the lattice constant; *W*(***r***) is the out-of-plane displacement of the plate; *u_j_* is the vibration displacement of the resonator; and *K_j_* and *m_j_* are the elastic stiffness and mass of the resonator, respectively. The material properties and dimensional parameters of the acoustic metamaterial plates are listed in tables S1 and S2. The plane wave expansion method is used to solve Eqs. 1 and 2, and the acoustic frequency–wave number band dispersive structure is obtained, as shown in fig. S8A. The gray area is the bandgap range of the acoustic metamaterial structure in out-of-plane (*z* direction) mode (*31*, *32*), and the red line marks the defect band. The defect band frequency is designed to be near 475 Hz, which is around the international standard pitch (440 Hz) (*33*) for better coverage of daily sounds. Considering acoustic wave excitation (*22*), the governing equation of the 2D local resonance metamaterial plate is

$$\begin{array}{c} {D\nabla^{4}W(r)-\rho h\omega^{2}W(r)=p_{\text{inc}}(r,z)|}_{z=0}+p_{\text{ref}}(r,z){|{}_{z=0}-p_{\text{tr}}(r,z)|}_{z=0}\\ +\sum_{j=1}^{N}\sum_{R}f_{j}(r_{j}+R)\delta [r-(r_{j}+R)] \end{array}$$ (3)

where *p_m_*(***r***, *z*)∣_*z* = 0_, with *m* = inc, ref, and tr representing the incident, reflected, and transmitted sound pressure located in position *z* = 0, respectively. The wave vector *k* = (*k_x_*, *k_y_*), *k_x_* = *k*_0_sinθcosφ, *k_y_* = *k*_0_sinθsinφ, and *k_z_* = *k*_0_cosθ, in which θ and φ are the elevation angle and azimuth angle of an incident sound pressure, respectively, and *k*_0_ = ω/*c*_0_ is the wave number. Equations 2 and 3 are solved to obtain the vibrational displacement field of the structure, and this field of the defect mode inside the bandgap is shown in fig. S8B. Around the defect mode frequency, incident sound waves are confined into the defect with amplified pressure, generating an electrical signal caused by piezoelectric coupling of the attached lead-zirconate-titanate (PZT-5H) thin film at the defect position. In text S1, we describe the derivation process of the piezoelectric energy conversion, with the generated output voltage and electric power as a function of load resistance shown in fig. S8C.

We then simulate the acoustic dispersion relation of the defected planar acoustic metamaterial plate for one face of the MBAS by applying the Bloch periodic boundary conditions, as shown in Fig. 1B. A pseudo-bandgap appears because of the large thickness-to-lattice constant ratio of the aluminum plate, whereby only two in-plane modes with a high phase velocity and a defect mode with out-of-plane vibration at about 488 Hz exist inside the bandgap, confirming the above analytical calculation. The vibrational profiles of these two in-plane modes and the out-of-plane defect mode are confirmed using simulated movies, as shown in Fig. 1B on the right side and movie S1. It shows that the vibration of the defect mode is strictly localized inside the cavity because of the forbidden bandgap for out-of-plane motions. To illustrate the origin of the outstanding sensitivity and SNR with omnidirectional beamforming, we simulate the spatial sound pressure field for three types of receiving devices, including the whole structure of the MBAS when the sound incident is normal to the top plane (plane no. 1 of the MBAS, along the *z* axis), as shown in Fig. 1C. Previous work demonstrated that a bare plate with PZT can harvest sound energy only under a weak transduction rate and with no source tracking (*21*, *22*). Although a spherical PZT array can achieve omnidirectional beamforming, it is still limited to a weak sensitivity because of rapid sound dissipation in space. Here, by combining planar acoustic metamaterial plates and a spherical arrayed structure, we achieve both strong pressure amplification where the root mean square (RMS) sound pressure is more than twice of the source and omnidirectional sound beamforming for all incident directions. In fig. S21, we show that the transmission spectra of the metamaterial defect cavity can be much higher than a bare plate without metamaterials. The enhanced transmission is about six times brought by metamaterials. This wave-confining property will notably improve sensitivity because of enhanced sound pressure at the PZT patch, while still keeping the ultralow self-noise of piezoelectric transducers, leading to a superior SNR.

### Sensor characterization

**Fig. 2.**
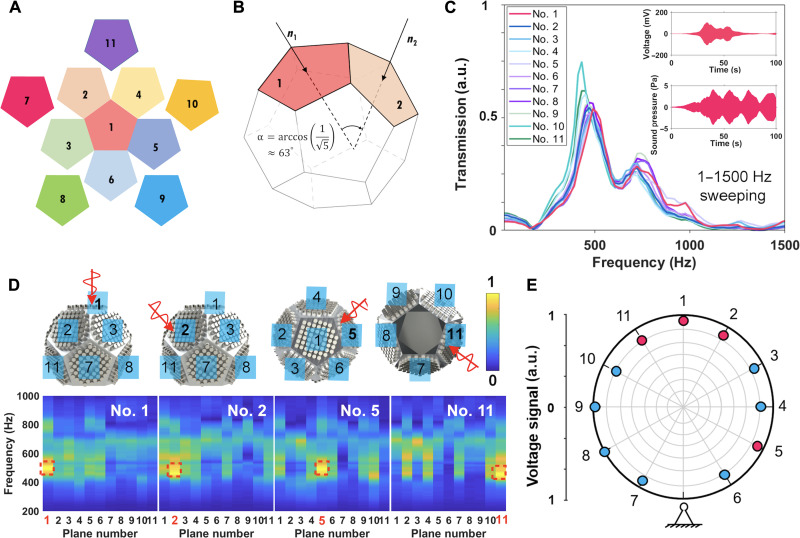
Regular dodecahedron structure and spatially omnidirectional beamforming characteristics of the MBAS. (**A**) The tile view of the MBAS, consisting of 11 planar local resonance acoustic metamaterials (known as the supercell) at its 11 facets, while the bottom facet is left empty for installation onto support structures. (**B**) The schematic diagram of the relative angle between two neighboring facets of the MBAS structure. (**C**) The transmission spectrum of the 11 supercells under normal sound incidence with respect to each of them. The insets exhibit the open-circuit voltage and the incident sound pressure measured by plane no.1 in the time domain. (**D**) The normalized signal maps of the MBAS, obtained when the 488-Hz sound is incident normal to four representative planes (no. 1, 2, 5, and 11). The red dashed boxes indicate that the plane facing the sound source shows the strongest response at the resonance frequency. (**E**) The received signal of the 11 planes of the MBAS showing spatially omnidirectional patterns, with the red color corresponding to the four planes shown in (D). a.u., arbitrary units.

The spherical surface of the MBAS is approximated by a regular dodecahedron structure with 11 facets for acoustic sensing and the last plane left empty for mounting. Figure 2A is the tile view of the plane positions for the MBAS structure. The top plane, opposite to the mounting facet, is named plane no. 1, while planes no. 2 to no. 6 are adjacent to the five sides of plane no. 1 to form a rim at the position of the second layer, and the third layer consists of planes no. 7 to no. 11, which connect with the planes on the upper layer. The relative angle, also known as the supplementary of the dihedral angle of a regular dodecahedron, between the adjacent planes (Fig. 2B) is defined as

$$\alpha =\text{arccos}(1/\sqrt{5})\approx 63{}^{\circ}$$ (4)

In text S2, we give more comprehensive sets of essential parameters and the topological model, including but not limited to its Schläfli symbol, volume, and midradius. Typical transmission spectra of planes no. 1 to no. 11 in the MBAS under normal incident acoustic waves ranging from 1 to 1500 Hz separately are shown in Fig. 2C, with the captured time-domain voltage and sound pressure data in the insets. All 11 transmission spectra show peaks at about 488 Hz, where the simulated defect mode is located, and also a wide frequency band instead of just at the peak frequency due to the low quality factor (*Q* ≈ 4.48) of the resonant cavity. There are also more peaks in the spectra due to multiple vibration modes of the aluminum plates (fig. S9), widening the frequency response, which is beneficial for the coverage of wide human phonetic frequencies (*34*). At the same time, the frequencies are amplified in the narrower bandgap that is specially designed to target the most relevant frequencies for daily conversations. Note that the 11 planes show slightly different spectra because of manufacturing deviation (see the “Experimental setup” section in Materials and Methods and fig. S10).

We also obtain the response of all other planes when the sound incidence is normal to one specific plane and exhibit their spectra from 200 to 1000 Hz, as mappings for four representative directions (normal to planes no. 1, 2, 5, and 11) being shown in Fig. 2D (more mappings are available in fig. S11). The incident sound pressure excites all 11 cavities in the MBAS from each direction normal to a plane of the dodecahedral sphere separately. Furthermore, the maximum signal always appears at the plane directly facing the sound source and at the resonance frequency, revealing that the MBAS is able to efficiently capture sound from different incident angles and discern the source angle by simply analyzing the signal intensity mapping. Moreover, the MBAS divides the whole space into 12 sections by the angle α as itself being the center, and the normalized signal received at each closest plane to the source is shown as a circular response pattern in Fig. 2E. This verifies its similar sensitivity and omnidirectional coverage of incident sound from a panorama of angles, which is beneficial for high-quality beamforming processing.

**Fig. 3.**
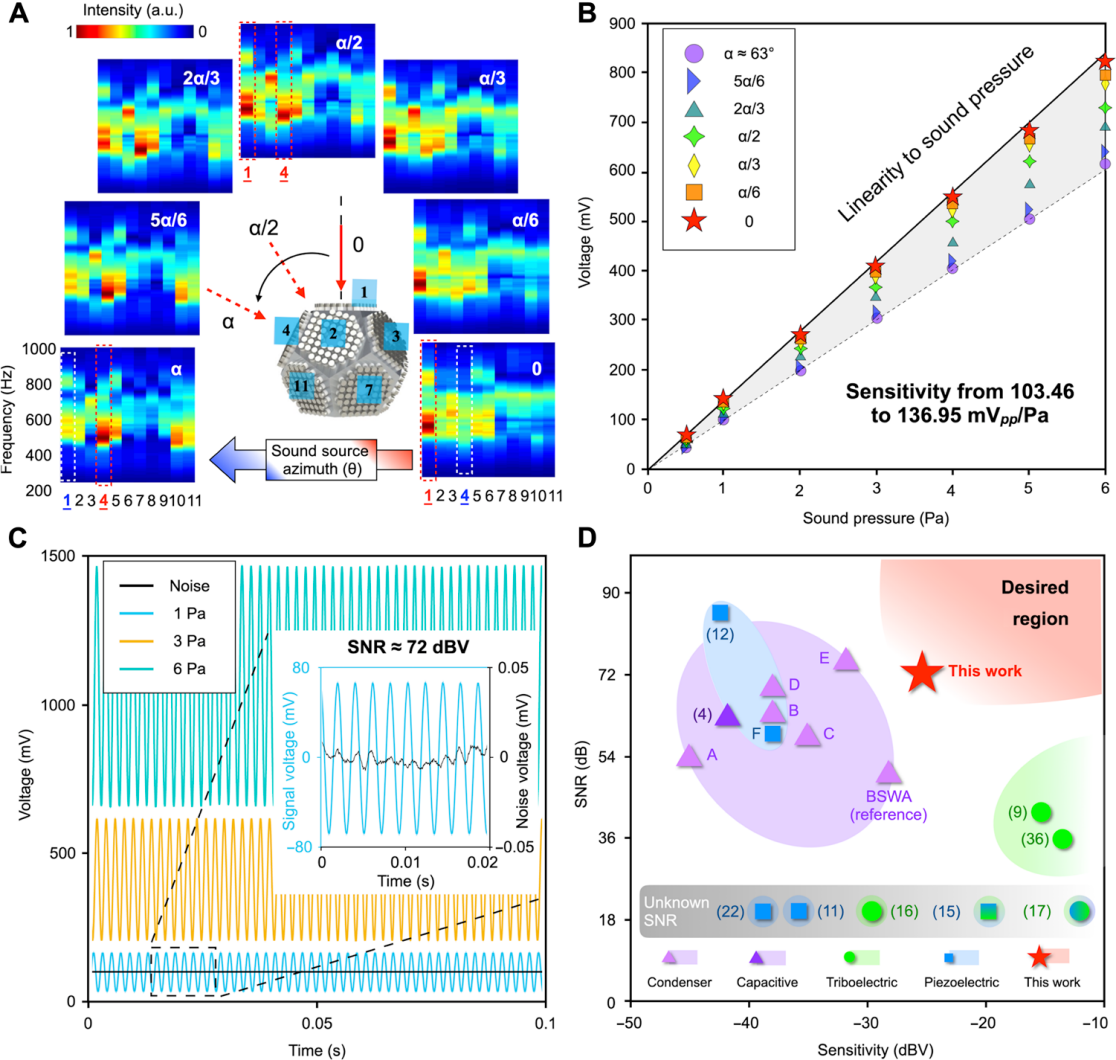
Characterization of the MBAS for source azimuth, sensitivity, and SNR. (**A**) The normalized plane-frequency signal maps of the MBAS, with different sound source azimuth, which gradually changes from 0 with respect to plane no. 1 to an angle of α with a step size of α/6. The inserted dashed boxes demonstrate that the response of plane no. 1 gradually weakens, and at the same time, the response of plate no. 4 gradually strengthens. (**B**) Peak-to-peak voltage of plane no. 1 as a function of sound pressure, with different sound source azimuth. (**C**) Voltage outputs of plane no. 1 as a function of sound pressures at 1, 3, and 6 Pa. The inset shows the zoom-in response at no input (in black) and at a 1-Pa pressure (in blue), which is used as the standard sound pressure (equivalent to 94 dB SPL) for calculating the SNR. (**D**) The intrinsic SNR (in decibels) and sensitivity (dBV) of the MBAS without beamforming enhancing algorithm are plotted and compared with existing acoustic sensors for various transduction principles, including piezoelectric, triboelectric, and capacitive, in different colors. The unified formula (Eq. 5) is adopted to convert all reported metrics. The commercial microphones listed in these figures are as follows: A, PUI Audio (POM-1345P-C3310-R); B, Knowles (SPU0410LR5H-QB); C, Panasonic (WM-61A); D, Invensense (ICS-40720); E, Primo (EM258); and F, Vesper (PMM-3738-VM1000-R) and BSWA (MPA231) as a reference.

To further characterize the omnidirectional source tracking and beamforming processing, we refine the amount of change in source angle by gradually modifying the incident angle with respect to plane no. 1 from 0 to the angle of α (≈63°, normal to the adjacent plane no. 4), shown as the sound source azimuth in Fig. 3A. The normalized plane-frequency mappings of the MBAS as a function of source azimuth are used to study their relationship. If the sound source is facing toward plane no. 1 (a 0° angle), plane no. 1 has the highest intensity (in red), while the plane with an angle of α° shows a weaker intensity. It indicates that the plane with the strongest intensity gradually shifts from plane no. 1 to plane no. 4, while they show almost equal intensity when the azimuth is α/2 (in the middle between planes no. 1 and 4). The strongest response switches to plane no. 4 when this angle reaches α. The results verify that the normalized signal maps of MBAS are directly associated with the direction of the sound source, which reflects the ability of MBAS to determine the source angles using the signal intensity of arrayed receivers.

We then measure the voltage output of plane no. 1 in the MBAS under resonance frequency, while the sound pressure varies from 0.5 to 6 Pa and the incident angle (source azimuth) varies from 0 to α. The peak-to-peak voltage shows a highly linear relationship as a function of pressure, as shown in Fig. 3B. The average sensitivity reaches an outstanding level at about 137 mV_pp_/Pa, or equivalently 48.4 mV_rms_/Pa or −26.3 dBV, without an external amplifier or beamforming enhancing algorithms. We have unified the calculation formula of sensitivity and provide the corresponding conversion methods here among different reports in literature

$$\begin{array}{c} \text{Sensitivity}=\frac{2\sqrt{2}∙∆V_{s}}{∆P}(mV_{\text{pp}}/\text{Pa})=\frac{∆V_{s}}{∆P}(mV_{\text{rms}}/\text{Pa})\\ =20\;{\text{log}}_{10}(\frac{∆V_{s}/∆P}{V_{0}/P_{0}})(\text{dBV}) \end{array}$$ (5)

where ∆*V*_s_ is the RMS of the output voltage, in which mV_pp_ and mV_rms_ are the units in peak-to-peak and RMS, respectively; ∆*P* is the sound pressure range; and *V*_0_ provides the reference voltage (1 V) at the reference pressure *P*_0_ (1 Pa) defined as 0 dBV. Some prior reports often offer peak-to-peak or amplitude values for calculating sensitivity, while commercial microphones are generally labeled with RMS values and in dBV, which we also use to compare with commercial sensors. The sensitivity of the MBAS is the highest among piezoelectric acoustic sensors to the best of our knowledge, excluding those combined piezoelectric-triboelectric sensors, which are actually dominated by the triboelectric transduction, as the latest result suggests (*15*, *17*, *35*, *36*). Because the sound pressure near the sensor is captured out of the wave-confining defect but at the same distance from the source, we believe that this superior sensitivity can be attributed to the enhanced sound pressure and cavity vibration inside the defect, consistent with the simulated pressure enhancement. In addition, the sensitivity of plane no. 1 varies between 103 and 137 mV_pp_/Pa as the incident sound angle changes from 0 to α, which is also helpful for sensitivity-adjustable scenarios for different applications. Under the condition of far-field measurement [the distance of the sound source is more than a reference boundary (*r*); details in text S8], we choose to use the signal from a high-sensitivity channel, which is the one directly facing the sound source. However, in near-field measurement, a high sensitivity will amplify the noise or disturbance, and thus, we should use the channels on the back side of the metasphere with respect to the sound source (*37*). Combining Fig. 3 (A and B), we first distinguish the angle of the sound source through the mapping and then select the best channel to use according to the sound pressure level (SPL).

A high sensitivity alone is far from enough for high-performance sound sensing, as a high SNR is also critical to ensure the transduction quality. Figure 3C shows the output voltage of plane no. 1 for different sound pressures of 1, 3, and 6 Pa under the resonance frequency compared to the output under no input. The zoom-in output voltage at 1-Pa sound pressure (standard 94 dB SPL) in comparison with a noise voltage is presented in the inset. The SNR is obtained by SNR(dB) = 20 log_10_*V*_s_/*V*_n_, in which *V*_s_ and *V*_n_ are the RMS values of the signal (at 94 dB) and noise voltages, respectively. The SNR of the MBAS thus reaches about 72 dB at the resonance frequency. Compared with triboelectric auditory sensors, which are the only type of acoustic sensors showing a higher sensitivity than the MBAS (*9*, *36*), the MBAS shows a superior SNR owing to the low self-noise of piezoelectric materials. Therefore, we achieve a combination of ultrahigh sensitivity and outstanding SNR due to the wave-confining capability and the low intrinsic noise of the MBAS, leading to a unique advantage in sound transduction comparing to all other types of acoustic sensors (*38*). The region of desired specification can show this conclusion in a joint SNR-sensitivity plot in Fig. 3D, where the MBAS uniquely locates inside the desired region, while the SNR could be further improved by beamforming algorithms to suppress noise. This advantage can also be shown by measuring using a newly coined metric, sum of SNR and sensitivity, named the whisper-hearing figure of merit in fig. S2.

### Conference aiding system

The advantageous metrics of the MBAS enable a better robotic auditory system for many critical applications. Virtual conference and hybrid online-offline meetings have seen a burst of expansion during the past couple of years. It is now an everyday scenario to attend a virtual meeting with some online attendees inside a large conference room equipped with multiple fixed microphones or arrayed microphones in the center of a conference table. However, these microphones are typically unsatisfactory in transducing voices, and they cannot differentiate the incoming voice angle to track the speaker. The MBAS-based conference aiding robot is able to not only detect voices under a hypersensitive rate but also support the identification of different users through the function of source localization.

**Fig. 4.**
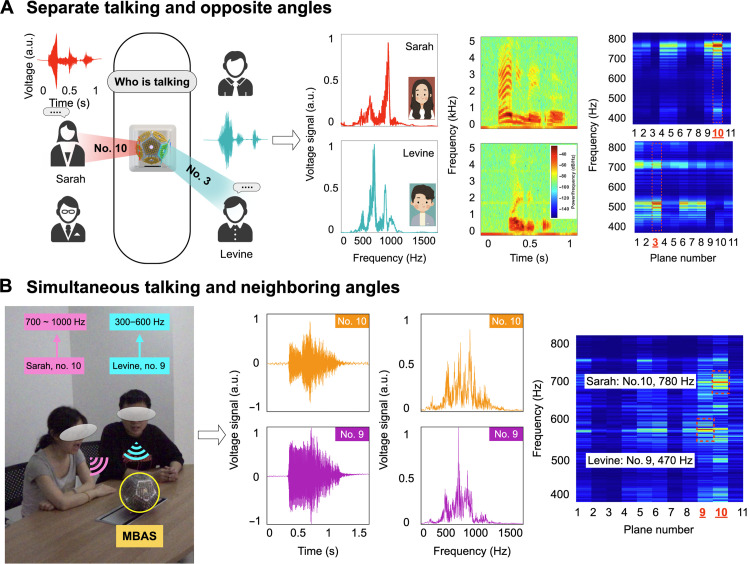
Applications of the MBAS in the conference aiding system. (**A**) Schematic diagram of the MBAS-based conference aiding system for separate talking and opposite angles, combined with FFT spectra, STFT spectrogram, and the normalized plane-frequency maps (Sarah in red, Levine in blue). (**B**) Experiment setup for simultaneous talking and neighboring angles, combined with FFT spectra, STFT spectrogram, and the normalized plane-frequency map (plane no. 9 in purple and no. 10 in orange, which indicates that the information of multiple sound sources is merged).

Specifically, two users (Sarah and Levine) are facing the opposite planes, saying the same word (for example, “elevator”) separately, and the corresponding signal processing took place behind the scenes. The MBAS captures the voice signals and generates a well-preserved electrical signal in the time domain, owing to its high sensitivity and SNR, good linearity, and broad frequency band, as shown in Fig. 4A. Then, fast Fourier transform (FFT) and short-time Fourier transform (STFT) methods are used to convert the voltage signal into the frequency domain, joint time-frequency analysis, and the normalized signal maps of the MBAS. It indicates that Sarah’s voice (female) is at a higher pitch, as the high-frequency components are more prominent. The joint time-frequency analysis also shows that Levine’s voice signal (male) is more concentrated in the low-frequency region than Sarah’s. Through the analysis of these signals, the identity of the speakers can be recognized. In addition, the normalized plane-frequency signal maps directly exhibit the sound source angle with respect to the MBAS as Sarah speaks to plane no. 10, while Levine speaks to plane no. 3, which adds another physical layer for speaker identification and tracking. More details are available in fig. S12. In addition, the Fourier transform for voice authentication can be completed in real time, as shown in movie S2.

Furthermore, we also performed experiments of the MBAS in a conference room scenario, including sounds with neighboring incident angles and sounds occurring simultaneously. In the first scenario as shown in fig. S20, Levine spoke first toward plane no. 9, shown as the signal in the first 2 s in the time domain. At the same time, the adjacent plane no. 10 also showed a smaller response. After that, Sarah spoke toward plane no. 10, and the voltage signal was within 2 to 4 s. As a result of processing by the MBAS, the source location and identity information could be distinguished by FFT (Levine, 500 Hz; Sarah, 700 Hz) and normalized plane-frequency signal maps (strong intensity in plane no. 9 at 500 Hz and no. 10 at 700 Hz, respectively).

In the second scenario as shown in Fig. 4B, in the case of simultaneous talking, especially with neighboring incident angles, the mutual interference between multiple sound sources is severe. This can be seen as neither the time-domain signal nor the Fourier transformed signal that can be effectively analyzed. Fortunately, as shown in the normalized plane-frequency signal map, we can distinguish that the two high-intensity responses occur at a relatively lower frequency in plane no. 9 and a relatively higher frequency in plane no. 10. This perfectly corresponds to Levine’s voice arriving toward plane no. 9 and Sarah’s voice arriving toward plane no. 10. This provides us a novel method of source tracking and authentication even when multiple sources are generating sounds simultaneously via the MBAS.

### Intelligent auditory system

**Fig. 5.**
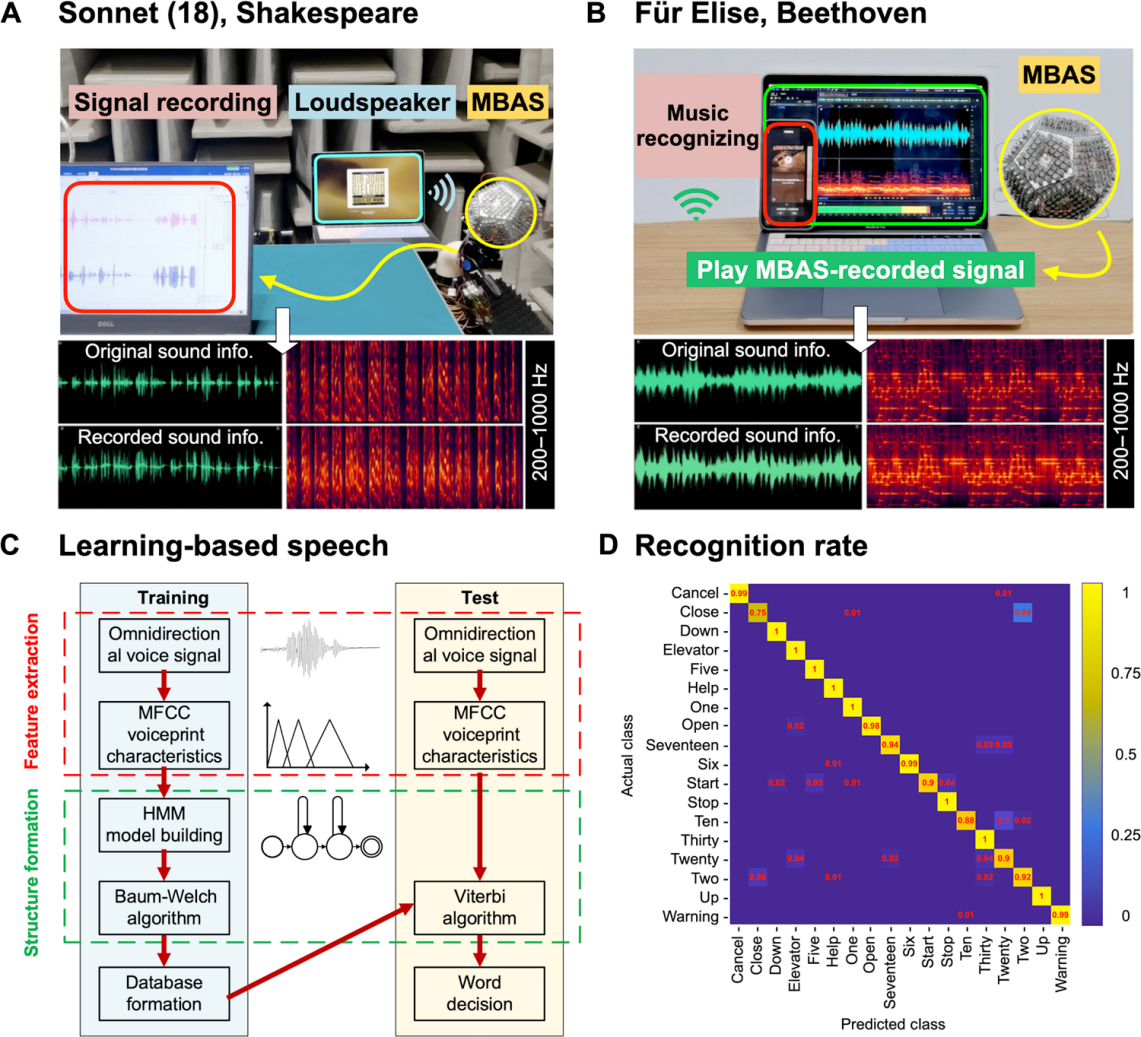
Machine learning–based speech recognition system of the MBAS with a high recognition rate. (**A**) Application of the MBAS-based auditory system used for recording while taking the Sonnet 18 as an example (relatively discrete, low-frequency voice). (**B**) Application of the MBAS-based music-recognizing system while taking the “Für Elise” piano music as an example (relatively continuous, wide-frequency voice). We show the original music wave and the recorded sound wave in the left bottom and the spectrograms of the original music and the recorded sound in the right bottom for both Sonnet 18 and “Für Elise.” (**C**) Flowchart of HMM algorithm for speaker training and testing procedures, composed of feature extraction and structure formation. (**D**) Classification test confusion matrix with 1980 groups of the dataset in recognizing 18 elevator instructions. The total classification accuracy can reach 96%. The color bar represents the accuracy of identification from 0 to 1.

We next use the MBAS to demonstrate its superior sound cloning capability and the high fidelity of the cloned sound. Our first example is a segment of Sonnet 18 read by a male at a relatively low pitch, as shown in Fig. 5A. This soundtrack features discrete and low-frequency sound compared to other continuous and wide-frequency music. We emphasize that no complex and power-consuming signal amplification or processing is involved, and we did not use any editing upon the recorded audio but instead display the waveform voltage output directly in the figure. The recorded sound is highly similar to the original sound, as proved by a comparison of the spectrograms between the original audio and the MBAS-recorded sound within 1000 Hz. More details are available in movie S3.

The second example is the piano music “Für Elise,” played by a computer loudspeaker and recorded by the MBAS (Fig. 5B). The time-domain data and frequency spectrum of the original and cloned music are presented without any signal processing or editing. It shows that the cloned music is highly similar to the original music in terms of time-domain audio and spectrograms. The recording and signal display of Sonnet 18 and classical piano music were performed in real time through the data acquisition device. To objectively show the high fidelity of the cloned audio, we use commercial music recognition software (CloudMusic, NetEase) to recognize the music recoded by the MBAS. It turns out that the recorded music by the MBAS can be correctly recognized in real time (within 5 s). More details are available in movies S4 and S5. In summary, the MBAS can perfectly clone both intermittent poem recitations and continuous piano music without any interfacing processing circuits and can successfully perform music recognition using commercial software.

We then perform speech recognition through machine learning. In text S3, we invite different people to give the pronunciation test using familiar words in daily life and then use the complete MBAS-based speech recognition and editing system to analyze the obtained data, from figs. S13 to S19. The response voltage signals of the MBAS exhibited scattering even in receiving the same word because of the uncertainty of pronouncing a particular term for the same person. Because of the influence of environment, emotion, and so on, the speech waveforms of speakers are quite different (*39*, *40*). The flowchart of the HMM (Hidden Markov Models)-based training and testing procedures for the MBAS-based robot is shown in Fig. 5C. We can offer feature extraction and model parameter formation on the voice signal input by the MBAS module and use the training program to record the voice information of the word in the database. This training part took a lot of time, which is an indispensable part of most machine learning applications. After training, 18 kinds of voice instructions can be recognized with a signal processing delay of less than 1 min. Given that mature commercial software exists for accurate speech recognition, we believe that a real-time recognition can be achieved using commercial algorithms but yielding much better sensing outcomes using our new acoustic sensor. Subsequently, a word decision is made by comparing the pretrained voice information with the test input. The training dataset (containing 1980 sets) of 18 word types are used to train the word classifier. The corresponding confusion matrix of the test dataset (containing 1980 sets, 110 sets for each type of word) for voice recognition is shown in Fig. 5D. We selected 18 groups of instructions, including actions (“Start,” “Stop,” and “Cancel”), numbers (“One,” “Two,” …, “Thirty”), voice-controlled equipment (“Elevator,” “Up,” “Down,” “Open,” and “Close”), alarm (“Warning”), and distress signal (“Help”) widely used in factory and also various buildings. As a well-known issue in the field, speech recognition accuracy is directly proportional to the number of speech training, thus making it more difficult to generalize the machine learning model with small datasets. Here, each plane of our adjustable-sensitivity MBAS is equivalent to a traditional microphone, leading to the fact that the voice information collected by the MBAS is 11 times that of other single microphones and that we can collect 11 sets of data at the same time. In the end, the overall recognition accuracy is close to 96%, and the effect of most words is immediate to or even 100%. This speech recognition application shows potential for creating a new speech recognition device for social robotics and rescue robots.

### Explore and rescue system

**Fig. 6.**
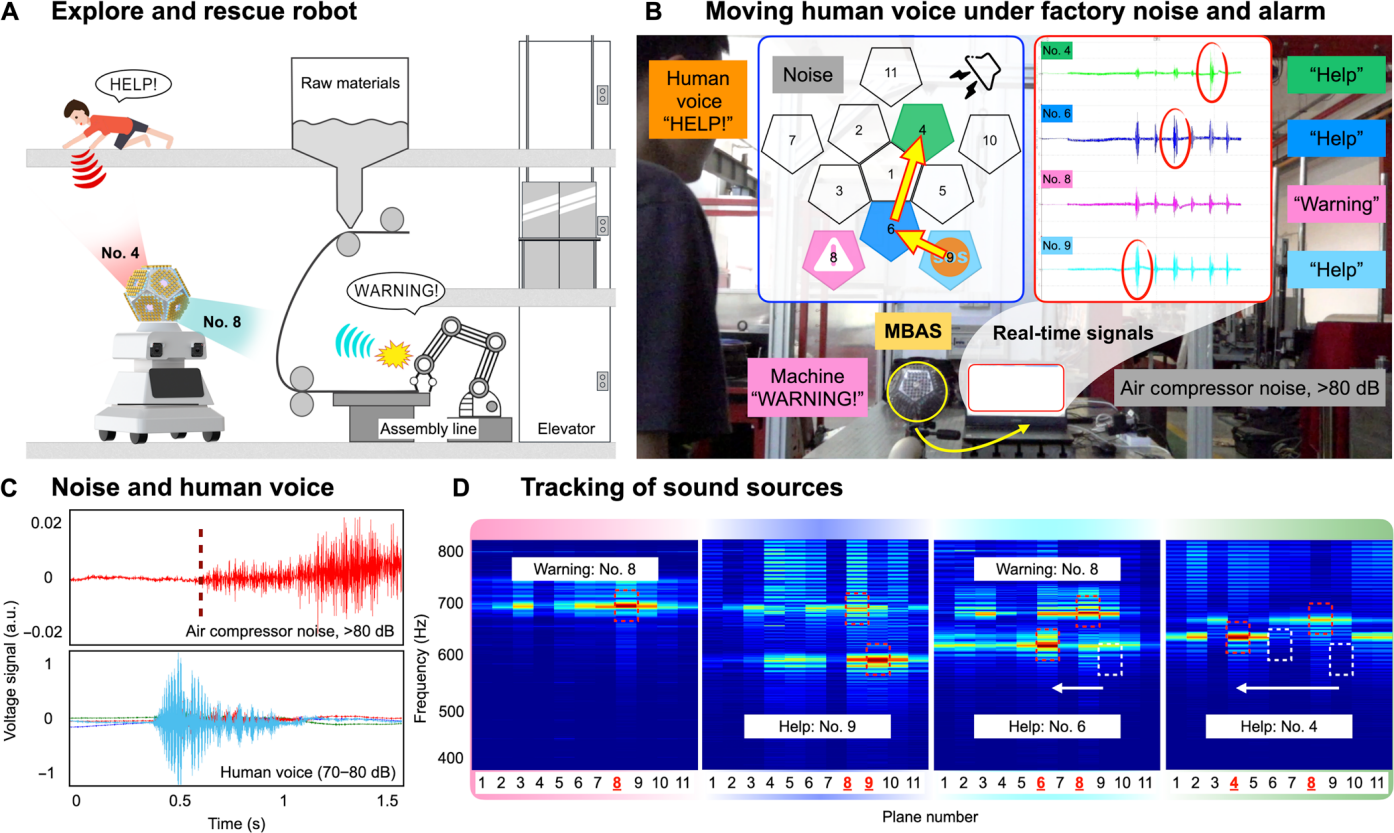
Applications of the MBAS in factory exploring and rescuing systems. (**A**) Schematic diagram of the MBAS-based factory exploring and rescuing robots. Speech recognition and sound source location can help with equipment failures or rescue in dangerous environments. (**B**) Experiment setup in a factory for multiple sound sources generating sound waves simultaneously at various incident angles. Background noises consist of environment noise, air compressor noise, and alarm. “WARNING!” is fixed (facing plane no. 8), and “HELP!” is moving from plane no. 9 to plane no. 4. (**C**) The voltage comparison between environment noise, air compressor noise, and the human voice. (**D**) The normalized plane-frequency maps to distinguish simultaneous “WARNING!” (plane no. 8) and “HELP! (planes no. 9, no. 6, and no. 4).

Driven by the development of Industry 4.0 and the Internet of Things, the high-end manufacturing industry is rising rapidly, where intelligent sensors and robots are highly desired. As shown in Fig. 6A, we visualize and prove that the MBAS-based rescue robot can be used for patrolling in a typical chemical factory filled with a multifloor material preparation system, rolling system, and product assembly system. Because of the complex images typically acquired inside a factory, sound information becomes a better source for rescue and surveillance. The advantages of the MBAS, such as adjustable sensitivity and high SNR, enable the robot to locate the sound source in the omnidirectional working space and identify critical signals. For example, the MBAS-based robot can capture a wounded person shouting for help and a dysfunctional machine sending out a warning signal.

In Fig. 6B, in addition to environmental noise, we have set up different sound sources to generate sound waves simultaneously, including (i) strong air compressor noise, (ii) machine alarm with repeating sound “WARNING!”, and (iii) a moving person yelling “HELP!” in different locations. Among them, the mechanical noise and the alarm were at opposite angles, and the person moved from an angle adjacent to the alarm (plane no. 8) (including the same height, plane no. 9, and different heights, plane no. 6) to the angle adjacent to the air compressor noise (plane no. 4). We plot the tile view of the MBAS to indicate the orientation of various simultaneous sound sources. The real-time signals are also displayed in Fig. 6B.

As shown in Fig. 6C, MBAS shows better sensitivity to human voices than lower-frequency noise, which makes it easy to recover the “HELP!” and “WARNING!” sounds overwhelmed by the heavy air compressor noise. Furthermore, as shown in Fig. 6D, we present the successful tracking of a moving sound source with normalized plane-frequency mappings, while the “WARNING!” sound is always facing plane no. 8. We demonstrate the tracking of the moving person yelling “HELP!” by an intense sound spot from plane no. 9 to no. 6 (similar angle to plane no. 8) and to plane no. 4 (similar angle to air compressor). Last, we again use the same machine learning algorithm to recognize the words under compressor noise in the factory, as shown in table S4.

Thus, we verify that in a real factory with intense background noise, MBAS can still complete the separation of multiple sound sources and the tracking of a moving human voice, with reasonable voice recognition based on machine learning algorithms. For the scenario that various sound sources generate sound waves simultaneously, even at similar incident angles, we believe that the MBAS can be regarded as a better voice-based HMI system in future intelligent machines.

## DISCUSSION

Currently, omnidirectional microphone arrays have been deployed in many sound reception occasions such as machine failure diagnosis, conference rooms, smart speakers, and robotic binaural auditory systems. To deal with complex spatial information, they usually consist of multiple MEMS microphones in various forms of geometric arrays, such as planar spiral arrays or 3D shelled arrays. Some of these systems have also adopted acoustic beamforming algorithms to further filter and track sound sources. Nevertheless, these acoustic systems are still limited by the intrinsic low SNR and sensitivity of their single microphone components. Although various advanced thin-film sensors, such as triboelectric sensors (*6*–*9*), have been developed for outstanding sensitivity, the advantage of their formation of a beamforming acoustic array is unclear. The spatial geometry of acoustic beamforming allows room for specially designed acoustic metamaterials, which are known for their sound manipulation capability (*18*, *41*). We thus show the first metamaterial spherical shell structure to omnidirectionally confine sound pressure in integrated piezoelectric defected cavities to harvest acoustic energy and record audio signal. Without affecting the acoustic beamforming structure, both the intrinsic SNR and sensitivity of single piezoelectric acoustic transducers are substantially improved at the same time. For future designs, we could combine adaptive stimuli-responsive materials (*42*, *43*) as the local resonators in metamaterials to actively tune the bandgap according to needs. Furthermore, by choosing appropriate materials, it is possible for MBAS to shrink in size with similar performance, extending the application fields to small-scale robotics and health monitoring (*44*–*46*).

In conclusion, we have demonstrated a metamaterial spherical surface with arrayed transducers in pressure amplification cavities for improved acoustic sensing and beamforming, proved by an outstanding SNR and sensitivity simultaneously, compared to other auditory sensors in Fig. 3 D and metamaterial-based acoustic sensing systems in table S3 (*22*–*27*, *47*, *48*). Its targeted broadband phonetic frequency response is specially designed for high-performance multifunctional verbal communication between humans and intelligent machines. This result promotes the HMI experience, energy saving, and easiness of interfacing processing circuits. We have also shown high-quality sound sensing and source tracking and a 96% speech recognition accuracy with machine learning, aiding the deployment of voice-based HMI ranging from conference assisting to daily surveillance.

## MATERIALS AND METHODS

### Sensor fabrication and assembly

The complete fabrication process of the MBAS includes the preparation of silicone rubber columns, assembly of the planar acoustic metamaterial plates, and 3D printing of the metasphere frame. The following steps achieve the preparation of the silicone rubber column. We first obtained uncured elastomer by mixing and blending the A/B parts of Ecoflex 00-30 (Smooth-On). After degassing in a vacuum oven (DZF-1AS, Kewei), we poured the Ecoflex gel into 3D-printed molds and scraped it evenly with a glass plate. To maintain the integrity of the silicone rubber columns, we sprayed a nonadhesive release agent (MS-122AD, Miller-Stephenson) beforehand. As the last step, they were cured at room temperature for 2 to 4 hours or in an oven at 40°C for 30 min, followed by demolding. We then marked the positioning grid on the aluminum plate by screen printing. In turn, the assembly of the silicone rubber columns, stainless steel columns, and the piezoelectric patch was completed to complete the fabrication of one complete plane. There are response differences between the 11 planes, which can be attributed to unavoidable manual fabrication errors. The key factor is that the frequencies corresponding to their prominent peaks are highly consistent, and therefore, it has little impact on speech recognition and sound source localization.

To fabricate the mounting frame, the STL (STereoLithography) file of the dodecahedral resin mounting bracket was exported from CAD (Computer Aided Design) drawing software (SOLIDWORKS, Dassault Systèmes SE) to a light-curing inkjet 3D printer (Objet500 Connex3 PolyJet 3D printer, Stratasys). Last, 11 planar acoustic metamaterial plates are assembled onto the frame to form the dodecahedron structure approximating a spherical surface. More details are illustrated in figs. S5 and S6.

### Simulation analysis

We simulate the characteristic frequencies, resonant modes, acoustic band diagram, and the frequency-voltage response diagram of the metasphere using the finite element software COMSOL Multiphysics. The simulation of the acoustic response for the MBAS is conducted by setting a 1-Pa incident sound pressure facing plane no. 1 and setting the azimuth to be 0° (normal to plane no. 1). We also set the air domain and perfectly matching layer around, which will absorb the sound wave reaching the boundary. The 3D model has directly imported from SOLIDWORKS-related links, and the material parameters are set according to table S1. When the device output voltage reaches peak response, the corresponding resonance frequency is 488 Hz, consistent with the defect frequency inside the acoustic bandgap.

### Experimental setup

The experimental setup includes sound signal generation, acoustoelectric signal conversion, and signal acquisition and processing devices (as shown in fig. S10). An arbitrary function generator (AFG3022C, Tektronix) generates oscillating signals, which are then amplified by a power amplifier (5016, SAST) before driving a speaker (SUINY). At the same time, a commercial microphone (MPA231, BSWA), placed at the same distance as the MBAS near the defect cavity, is used to measure the incident sound pressure as a reference. This sensor is positioned outside of the MBAS defect cavity so that it measures the typical air pressure after propagation over the same distance as the MBAS but without sound focusing and pressure enhancement. A data acquisition system with analysis software (DHDAS, Donghua) is used to record all data from the MBAS and the microphone simultaneously. We carried out the whole test in a semianechoic chamber, with a typical background noise at 15.6 dB.

## Supplementary Materials

### This PDF file includes:

Texts S1 to S8 Figs. S1 to S21 Tables S1 to S4 References
