# Supplementary material for: A wave-confining metasphere beamforming acoustic sensor for superior human-machine voice interaction
Source: Sci Adv. 2022 Sep 28;8(39):eadc9230. doi: 10.1126/sciadv.adc9230 (PMC9519046; doi:10.1126/sciadv.adc9230)
Supplement: Supplementary file 1 — Texts S1 to S8 Figs. S1 to S21 Tables S1 to S4 References [file sciadv.adc9230_sm.pdf]

Science Advances
AAAS

Supplementary Materials for

A wave-confining metasphere beamforming acoustic sensor for superior human-machine voice interaction

Kejing Ma *et al.*

Corresponding author: Lei Shao, lei.shao@sjtu.edu.cn; Wenming Zhang, wenmingz@sjtu.edu.cn

Sci. Adv. **8**, eadc9230 (2022)
DOI: 10.1126/sciadv.adc9230

The PDF file includes:

Texts S1 to S8
Figs. S1 to S21
Tables S1 to S4
Legends for movies S1 to S6

Other Supplementary Material for this manuscript includes the following:

Movies S1 to S6

Text S1. Theoretical analysis of planar acoustic metamaterials

We here theoretically calculate the converted electrical voltage from the strain field inside the defect cavity of the acoustic metamaterial plates. According to the elastic theory of thin plates, the strain along the *x* and *y* directions can be expressed as

$$S_x = -z\frac{\partial^2 W}{\partial x^2};\ S_y = -z\frac{\partial^2 W}{\partial y^2}; S_{xy} = -z\frac{\partial^2 W}{\partial x \partial y}. \qquad \textbf{(S1)}$$

Since the piezoelectric layer is very thin compared with the plate size, we assume that the stress along the *z* direction in PZT is zero. Therefore, the potential shift along the *x* and *y* directions is dx = dy = 0. The calculated output voltage and power of the planar acoustic metamaterial plate are shown in **fig. S8 C**, while ringing at the defect mode frequency, as a function of the external resistance from 1 Ω to 10 MΩ. The output voltage is seen to gradually increase to 48 mV and then maintains at this value. Simultaneously, the electric power gradually increases to the peak value of 0.023 μW, at this time, the resistance value is near 12.6 kΩ; after reaching the optimal resistance, the power value decreases gradually with the increase of the resistance.

Text S2. From planar metamaterials to a metasphere

The key parameters and dimensions of the metasphere are shown in **Table S1** and **S2**. The metasphere is approximated by using a regular dodecahedron (one of the five Platonic solids), which can be defined as {5, 3} with Schläfli symbol (a regular polyhedron having 3 pentagons around each vertex). The volume and surface area are

$$V = \frac{1}{4}(15 + 7\sqrt{5})a^3 \qquad \textbf{(S2)}$$

$$A = 3\sqrt{25 + 10\sqrt{5}}a^2 \qquad \textbf{(S3)}$$

where $a = 64\,mm$ is the edge length of a regular dodecahedron. Then, we define the circumscribed sphere is one that touches the regular dodecahedron at all vertices, and the inscribed sphere is tangent to each of the regular dodecahedron's faces:

$$r_u = a\frac{\sqrt{3}}{4}\left(1 + \sqrt{5}\right) = a\frac{\sqrt{3}}{2}\phi \qquad \textbf{(S4)}$$

$$r_i = a\frac{1}{2}\sqrt{\frac{5}{2} + \frac{11}{10}\sqrt{5}} = a\frac{\phi^2}{2\sqrt{3 - \phi}} \qquad \textbf{(S5)}$$

where r_u is the radius of a circumscribed sphere, r_i is the radius of an inscribed sphere, and ϕ is the golden ratio; while the midradius, which touches the middle of each edge, is:

$$r_m = a\frac{1}{4}\left(3 + \sqrt{5}\right) = a\frac{\phi^2}{2}\ . \qquad \textbf{(S6)}$$

Text S3. Daily words analysis of different people

In **fig. S13**, we invite different people to give the pronunciation test using familiar words in everyday life. As shown in **fig. S14**, the time-domain diagrams of three male and three female reading the same word "weather" are presented. We record the names of three female as M, G, and O, and the names of three male as C, L, and W. The voltage changing trend of the six data groups has similarities with time, but the amplitude details are different. The output signal amplitude of male is generally more significant than that of female.

As shown in **fig. S15**, we use fast Fourier transform (FFT) to transform the voltage signal into the frequency domain as a function of time. In FFT analysis, each person's signal distribution has two principal peak frequency distributions for the spectrum of the pronunciation of the same word. Still, the specific output signal varies with the frequency. We divide them into three groups according to similarity, as {M & C}, {G & L}, and {O & W}. Among them, the main peak frequency of the first group is between 600-800 Hz, and the sub-peak frequency distributes near 500 Hz; The main peak frequency of the second group is near 500 Hz, and the sub-peak frequency gets higher; Although the main peak frequency of the third group is also near 500 Hz, the sub-peak has similar amplitude with the main peak, which distributes between 600-800 Hz.

To sum up, due to each person's different pronunciation habits, sound quality, and personality types, the time length, amplitude, and frequency distribution of the final output signal are different. On the one hand, it is difficult to find the pronunciation characteristics of the same word through the simple comparison of time-domain and frequency-domain diagrams, which is also the difficulty of speech recognition; On the other hand, it shows that everyone's voice characteristics are different, which lays a foundation for identity recognition and information encryption.

Text S4. Daily words analysis of the same person in different situations

As shown in **fig. S16**, we also recorded the sound information of the same person reading the same word in different states. Take "Music" as an example. By comparing six groups of data in the time domain, we can find that the overall trend of the signal is generally the same, but the details are slightly different. For instance, due to emotional fluctuation, the volume of speech will change, reflected as the different amplitude of voltage signals (such as the first and fifth time). Due to the change of intonation, word stress will change, reflected as the different waveform of voltage signal (such as the first, second, and sixth times and the third, fourth, and fifth times). Due to the speed difference, the duration of word reading will change, reflected as the different lengths of the voltage signal.

As shown in **fig. S17**, we use the fast Fourier transform (FFT) to convert the time domain data to the frequency domain for analysis. It can be seen that the correlation between the voltage waveform and the resonance frequency is the largest. The first, second, and sixth times are a group, and the third, fourth, and fifth times are a group. The common feature is that the pronunciation frequency is basically within 1000 Hz, and the formant is concentrated chiefly at about 500 Hz, which is consistent with the performance of the MBAS.

Text S5. Elevator instructions analysis for remote control

As shown in **fig. S18**, we analyze the voltage response time domain of seven elevator command voice signals. The conclusion is that the number of peaks is related to the syllable of the word, the waveform contour is related to the syllable pronunciation, and the seven instructions are different.

As shown in **fig. S19**, after fast Fourier transform (FFT), the main peaks (the position of the maximum response voltage) of "Elevator", "Up", "Down", "Cancel", "One", "Six" and "Thirty" appear at 521 Hz, 788 Hz, 843 Hz, 457 Hz, 529 Hz, 375 Hz and 528 Hz respectively, and the corresponding secondary peaks (the position of the second largest peak voltage) At 792 Hz, 526 Hz, 498 Hz, 606 Hz, 703 Hz, 495 Hz and 352 Hz, respectively. Due to the broadband characteristics of the MBAS, the differences of seven groups of instructions can be effectively identified through high fidelity and high accuracy information acquisition. Then, we use the collected word information to establish a database that can not only assemble sentences through

cloning or editing, but also recognize sentences through machine learning. Finally, a complete set of illustrative sentences can be put into the intelligent social robot to help control the passenger elevator during the epidemic.

Text S6. Elevator instructions recognition for direction discrimination

As shown in **Fig. 2 D**, the acoustic metasphere has directional sensitivity. When the sound source is incident on a plane, the voltage at the resonance frequency of the plane is much higher than that of other planes. As shown in **Figs. 2 C and E**, the response voltage values of the eleven plates when sensing normal incidence are almost the same. Therefore, on the whole, this means that the whole space is acoustically isotropic. Based on these two characteristics, it can be used to judge the approximate orientation of sound sources in space.

As shown in **fig. S12**, in the elevator instruction recognition project, when Levine says "Elevator" to plane No.10, we record the time-domain waveforms and frequency components of the response voltage of all planes. The response voltage of the eleven planes has the same trend with time, but the amplitude of the time-domain waveform is quite different. The response voltage of plane No.10 is the largest, and that of plane No.3 is the smallest, which is closely related to the spatial structure of the device. Then we can judge that the sound source is in the area in front of plane No.10, although the incident angle cannot be precisely determined. After that, we use the fast Fourier transform (FFT) method to convert the voltage signal as a function of time to the frequency domain, and obtain the same conclusion.

Text S7. Comparison for metamaterial-based acoustic sensing systems

In **Table S3**, we list the previous metamaterial-based acoustic sensing system. Through some indicators (such as sensitivity, signal-to-noise ratio, etc.), we compared them with our work, and clarified the innovation.

Most previous acoustic sensing systems enabled by metamaterials do not provide its sensitivity or signal-to-noise ratio, as they typically only used commercial microphones and emphasized on the physics of sound manipulation or enhancement using new metamaterials. In this work, our sound detection method is based on the significantly enhanced resonance of embedded metamaterial defect cavities, rather than relying on commercial microphones used in previous literature. This new design combines the all-directional sound signal enhancement brought by the metasphere and the low self-noise of piezoelectric sensing cavities. Therefore, it shows both outstanding sensitivity and signal-to-noise ratio, comparing to all other acoustic sensors.

Text S8. Reference boundary distance for different sound field models

According to the distance between the sound source and the microphone array, there are two models (near-field and far-field) with a reference boundary:

$$r = 2L^2/\lambda = 2fL^2/c \quad \textbf{(S7)}$$

, where r is the reference boundary distance, L is the array dimension, $\lambda = c/f$ is the operating wavelength, c is the wave speed, and f is the resonance frequency.

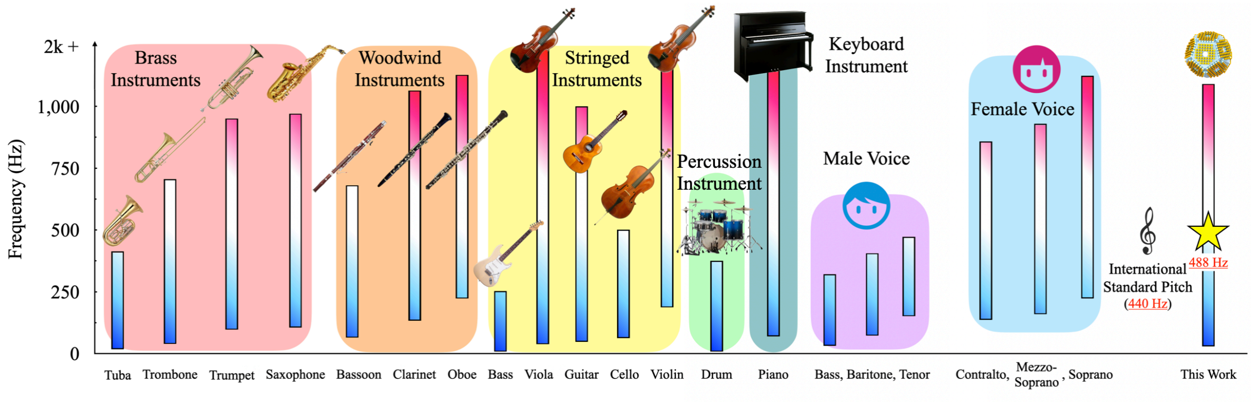

fig. S1. Various sound types and their corresponding frequency ranges.

The most common sound types including popular musical instruments (such as guitar, trumpet, saxophone, etc.), male and female voices show their frequency ranges are between 200 Hz and 1000 Hz. The International Standard Pitch (at 440 Hz) and the frequency range of the MBAS in this work are also shown in the right side.

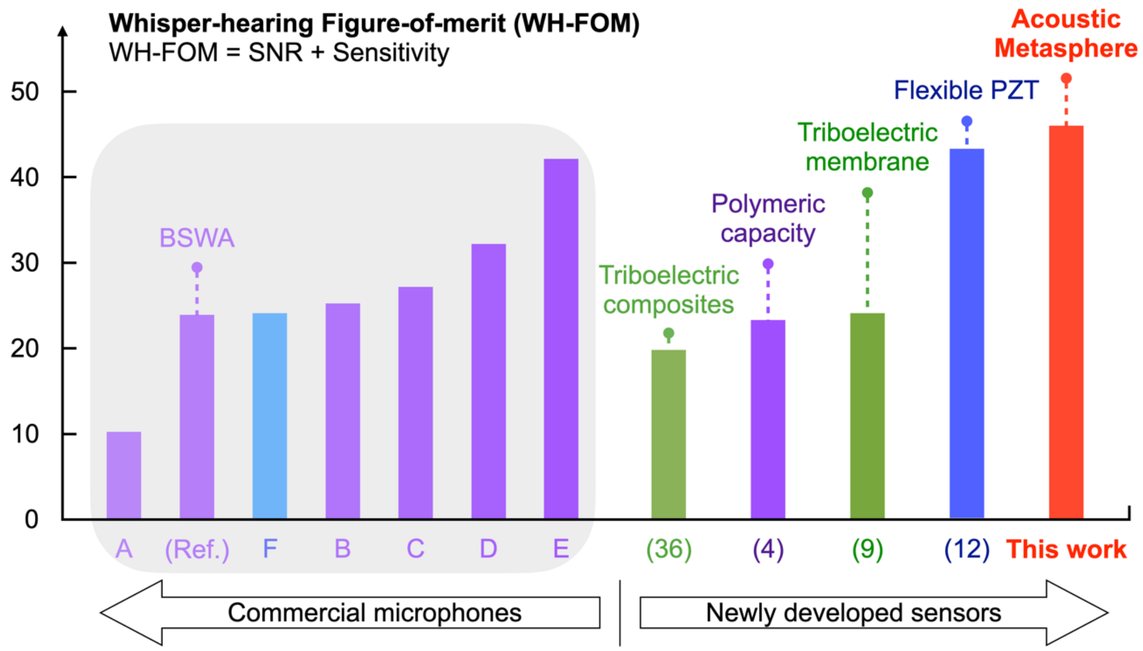

fig. S2. Whisper-hearing figure-of-merit (WH-FOM).

In order to clearly compare different acoustic sensors while promoting a high SNR and a high sensitivity simultaneously, we coined a new figure-of-merit for acoustic sensors, which is named as the whisper-hearing figure-of-merit (WH-FOM), as the sum of the SNR (in dB) and the sensitivity (in dBV). The WH-FOM values of each acoustic sensors shown in fig. S2 are plotted for comparison, including both commercial microphones and newly developed sensors in the literature. The sensor proposed in this work shows the highest WH-FOM as it is associated with both an ultra-high SNR and an outstanding sensitivity, implying its unique advantages in acoustic transduction.

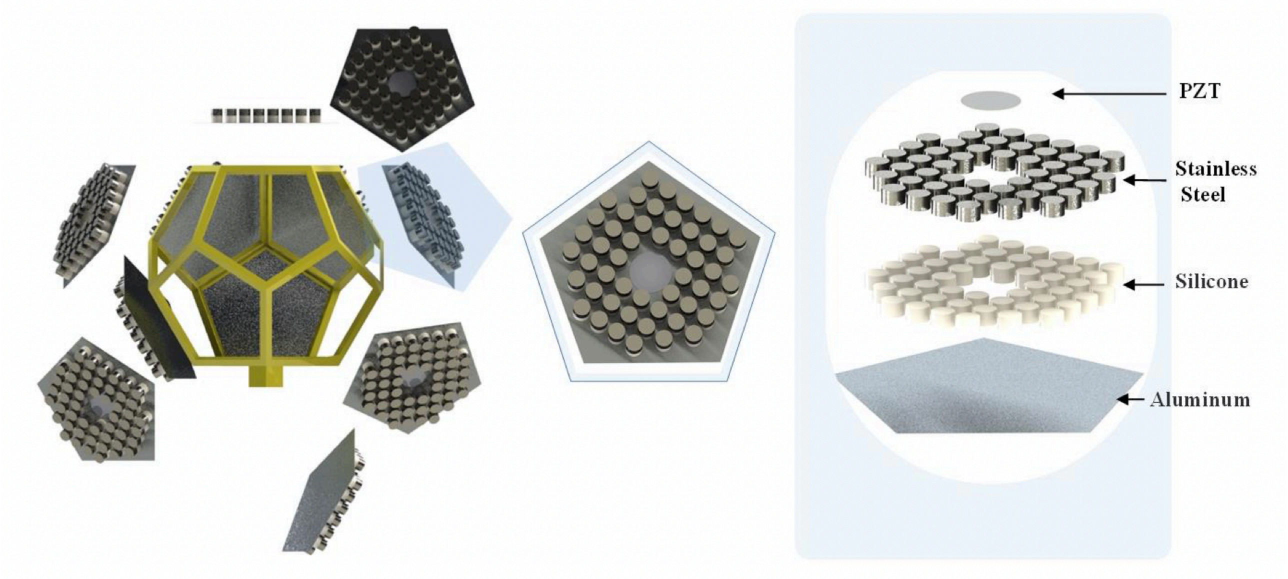

fig. S3. Exploded view of the MBAS.

The left figure shows the assembly structure, the middle figure is a single metamaterial plate, and the right figure displays the materials and shapes.

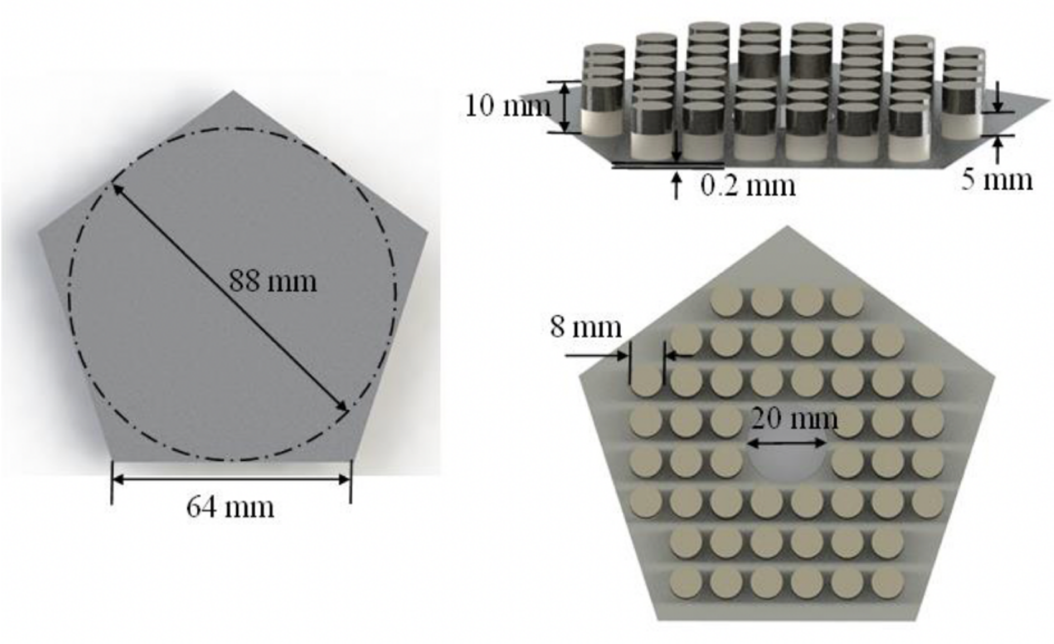

fig. S4. Dimensions of the acoustic metamaterial prototype.

To assemble a three-dimensional structure similar to the spherical shape, we choose the dodecahedron geometry consisting of 11 regular pentagon metamaterials.

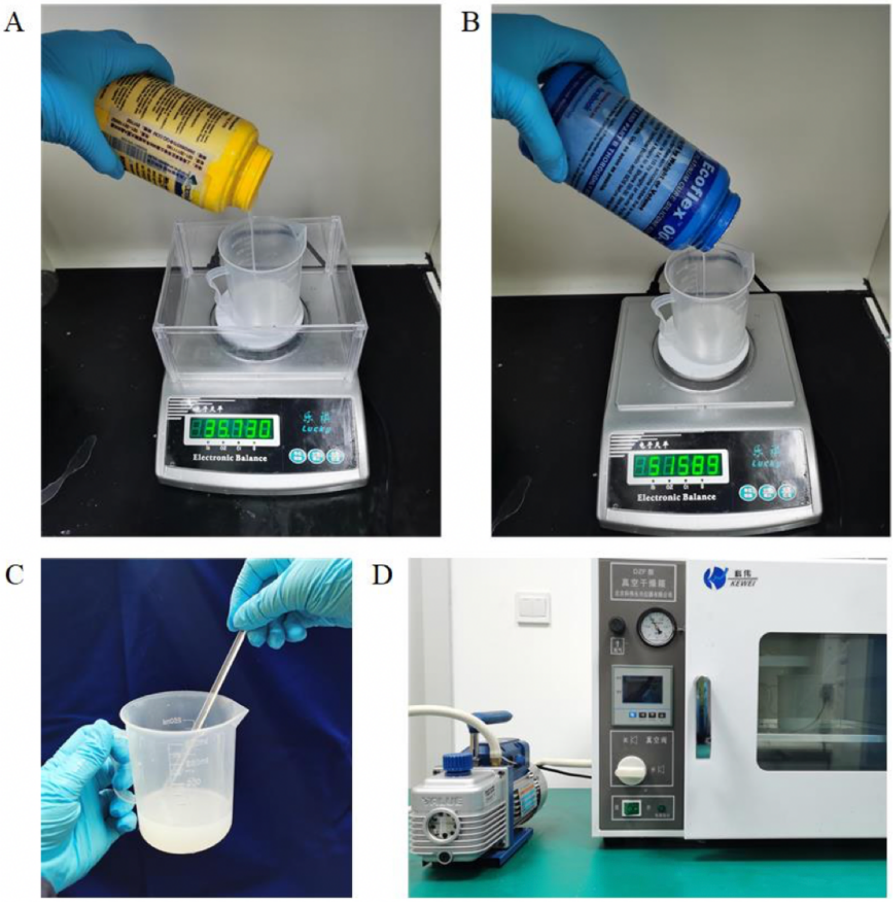

fig. S5. Fabrication of the silicone rubber columns.

(**A**) Weighing Part A and (**B**) Part B. (**C**) mixing the two parts by weight of 1:1. (**D**) debubbling in a vacuum oven.

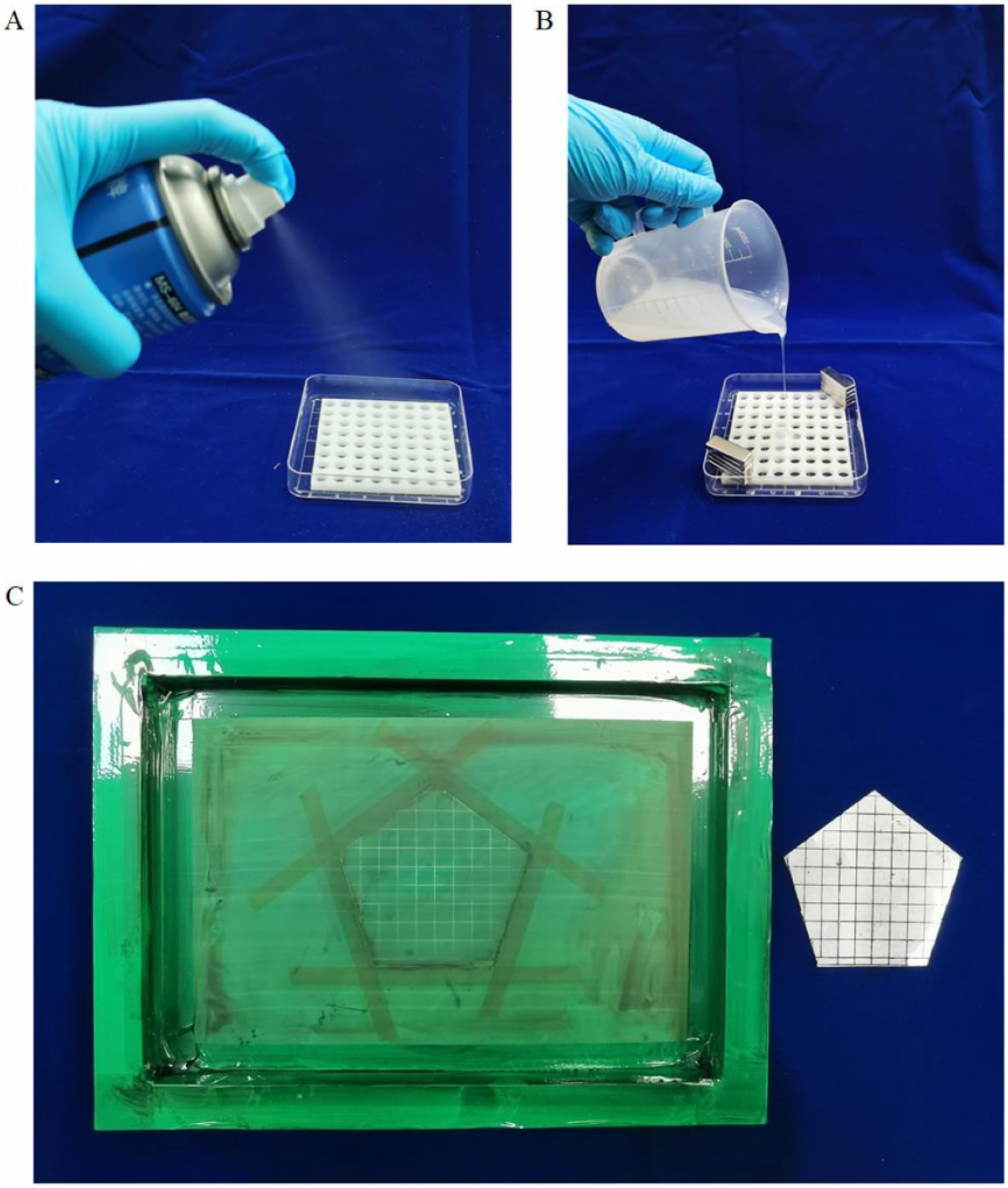

fig. S6. Assembling of a planar acoustic metamaterial plate.

(**A**) Spraying release agent onto the molds. (**B**) Molding process of the silicone rubber. (**C**) Screen printing on aluminum plate for the positioning of silicone rubber columns, which will be glued according to the printed mesh.

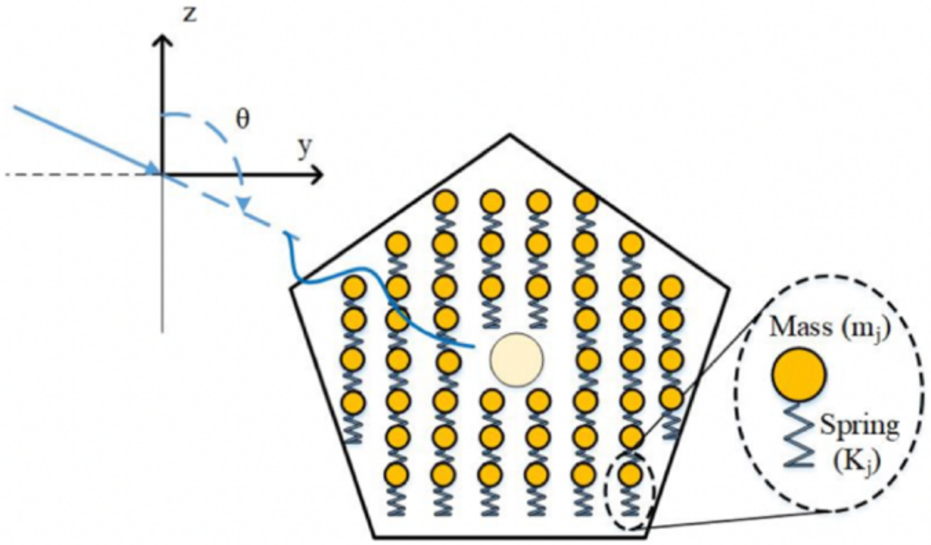

fig. S7. Schematic model of a pentagon-shaped planar acoustic metamaterial.
When the incident direction of the sound wave coincides with the normal direction of the plane, the incident angle of the sound pressure is 0°, which is called normal incidence.

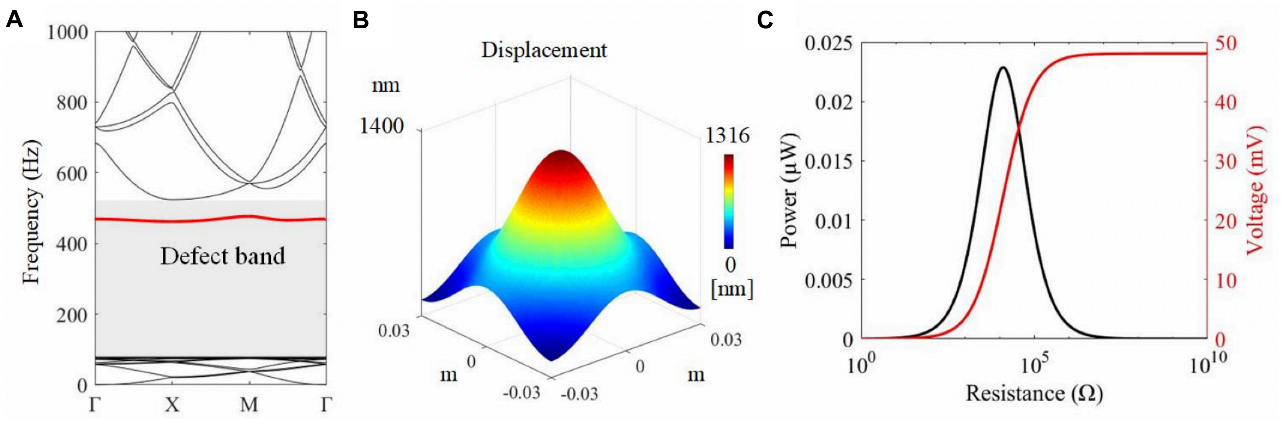

fig. S8. Theoretical analysis of a planar acoustic metamaterial plate.

(**A**) The band structure of the supercell for out-of-plane vibrations (along the *z*-direction) calculated from analytical theory. The gray area is the bandgap range, and the red line marks the defect band. The bandgap ranges from 79 Hz to 524 Hz, and the defect band frequency is 475 Hz. (**B**) The vibration mode shape of the defect mode when the incident sound wave is along the normal direction, showing the induced vibration is confined inside the cavity. (**C**) Electrical output performances with respect to the load resistance.

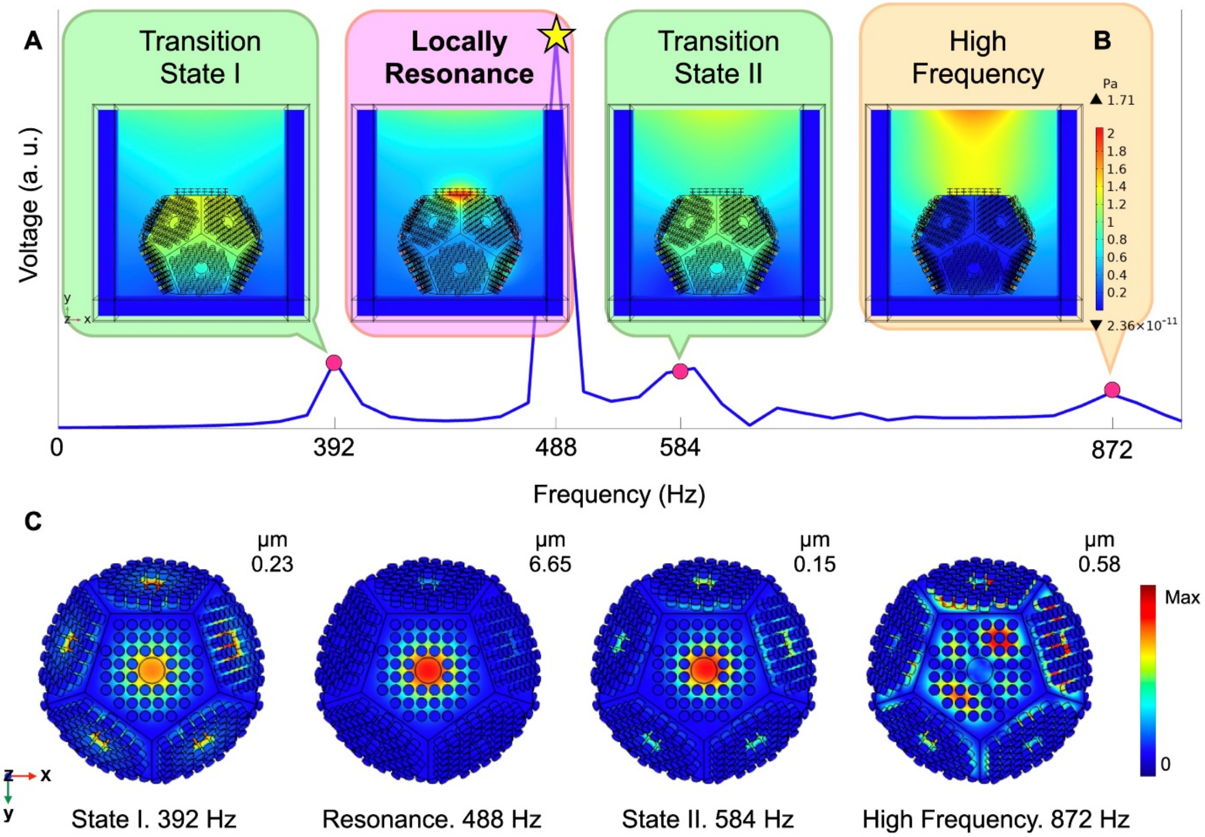

fig. S9. The simulated frequency response of the metasphere structure.

(**A**) The simulated open circuit voltage from 0 to 1 kHz. (**B**) The simulated sound pressure distribution at four peak frequencies. (**C**) The simulated vibration mode shape of the metasphere at the four corresponding frequencies.

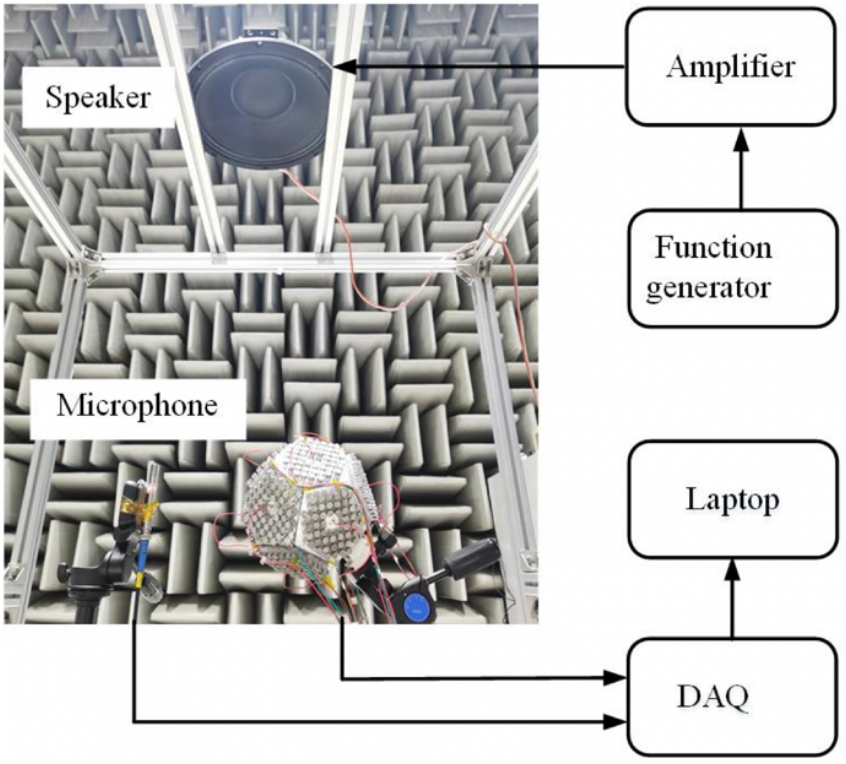

fig. S10. Experimental setup with schematic diagram of the signal acquisition.

A speaker and related equipments were used to generate sound waves of the specific frequency and pressure. MBAS was directly connected with a data acquisition equipment, without any other complex data processing (a commercial microphone was used as a reference).

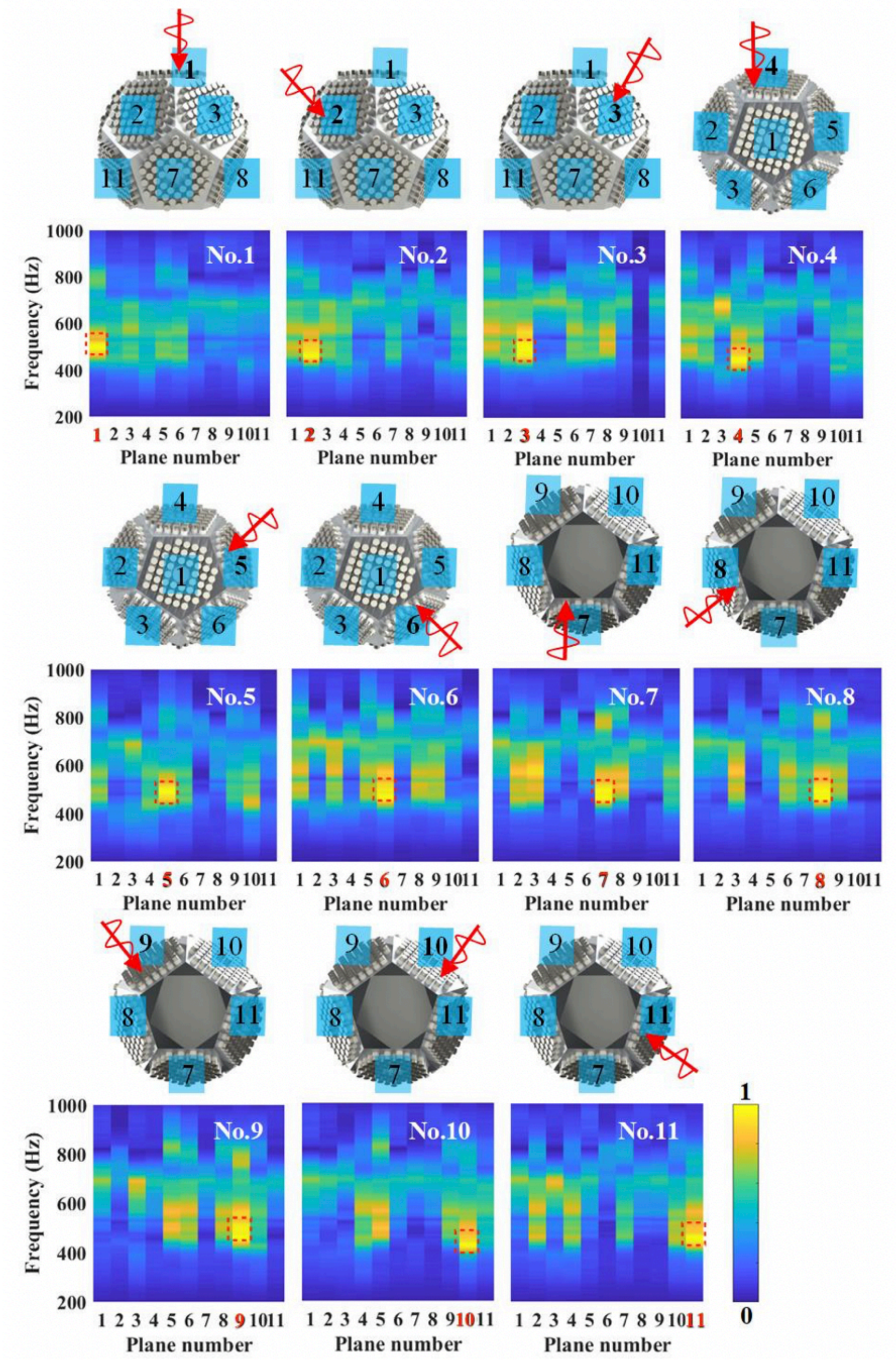

fig. S11. Normalized signal maps of MBAS with various incident angles.

A typical compilation of the normalized signal maps of MBAS corresponding to eleven incident angles normal to the eleven plates, respectively.

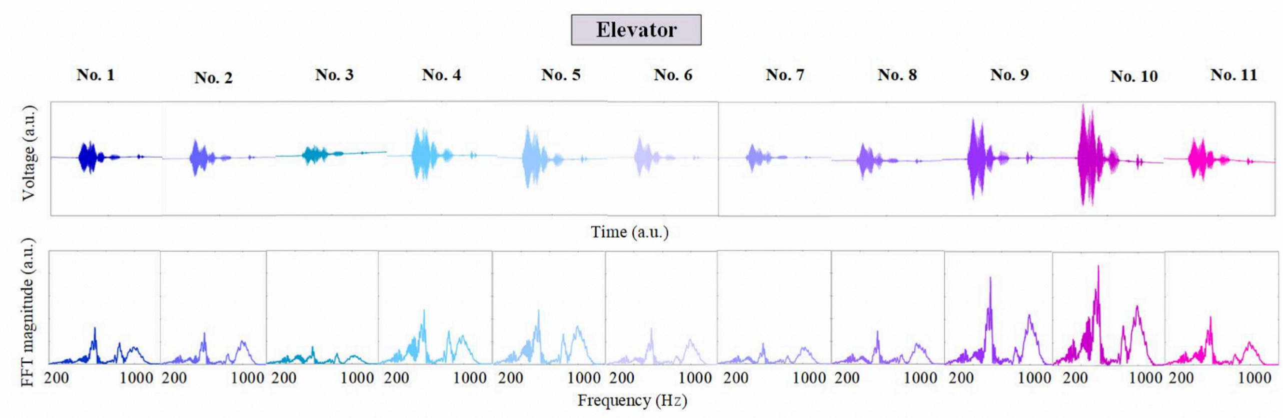

fig. S12. Temporal and frequency-domain signals of MBAS.

The response signal of the eleven planes in the time domain (top panel) with their respective FFT (bottom panel) while the voice incident normal to the plane No.10.

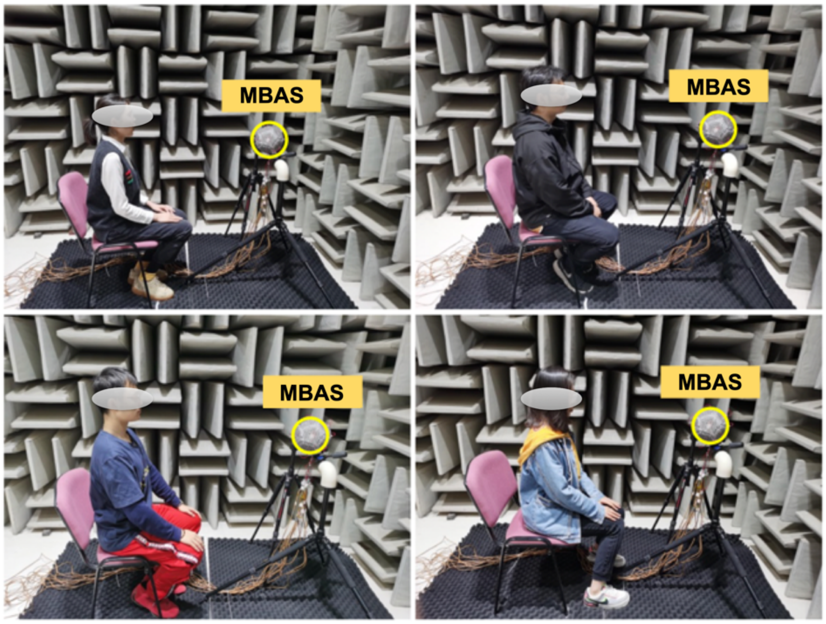

fig. S13. Experimental setup for daily words analysis.
Photographs of the voice recording and recognition experiment for four different people.

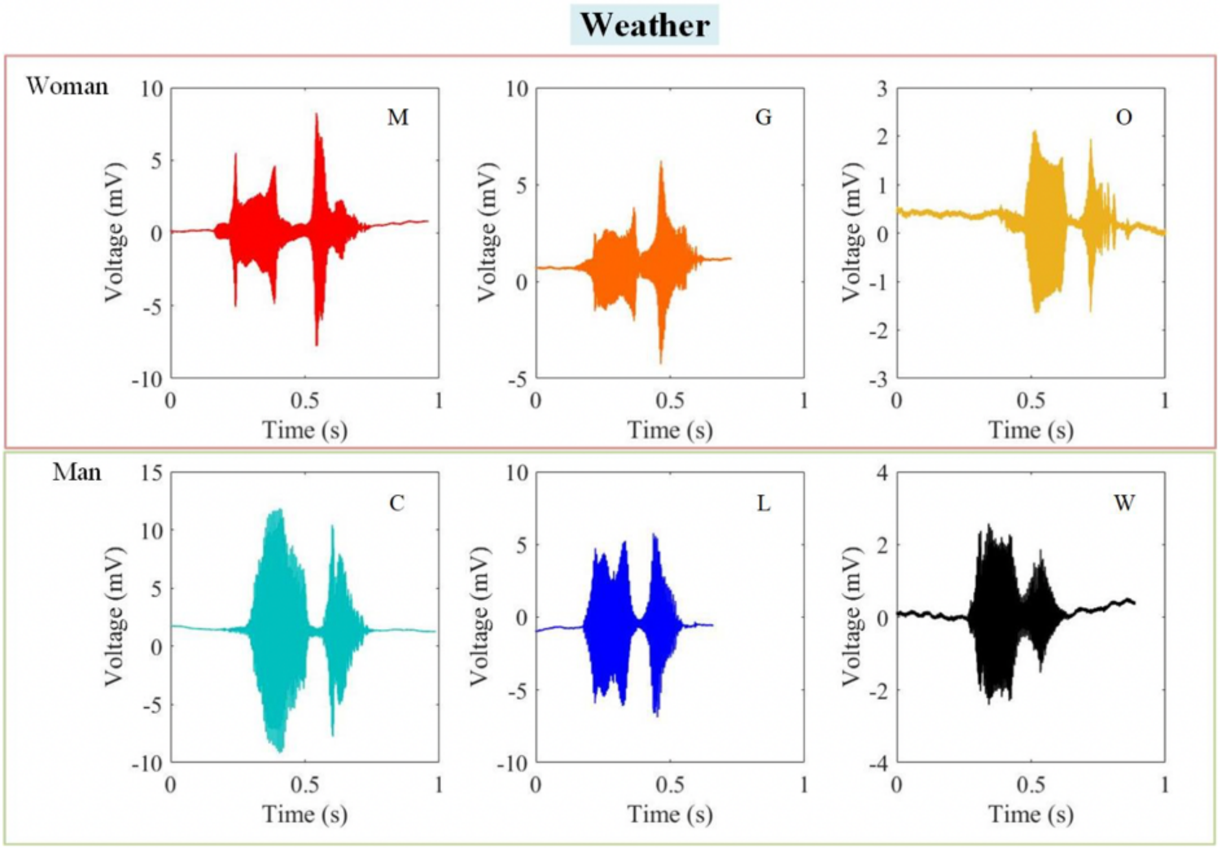

fig. S14. Temporal signals of the same word from six people.

Comparison of the MBAS-recorded time traces for the same word (“weather”) pronounced by three females (top panel) and three males (bottom panel).

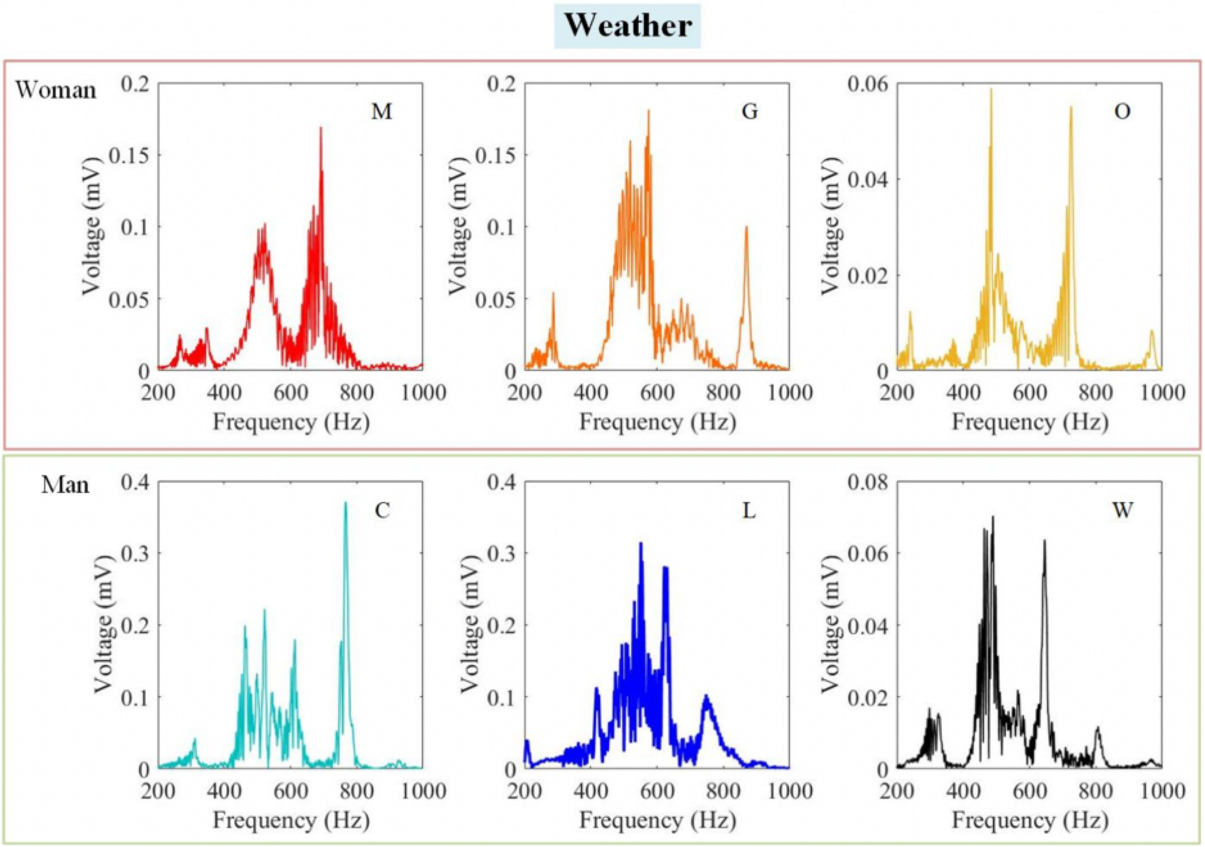

fig. S15. Frequency-domain signals of the same word from six people.

Comparison of the MBAS-recorded frequency responses for the same word ("weather") pronounced by three females (top panel) and three males (bottom panel).

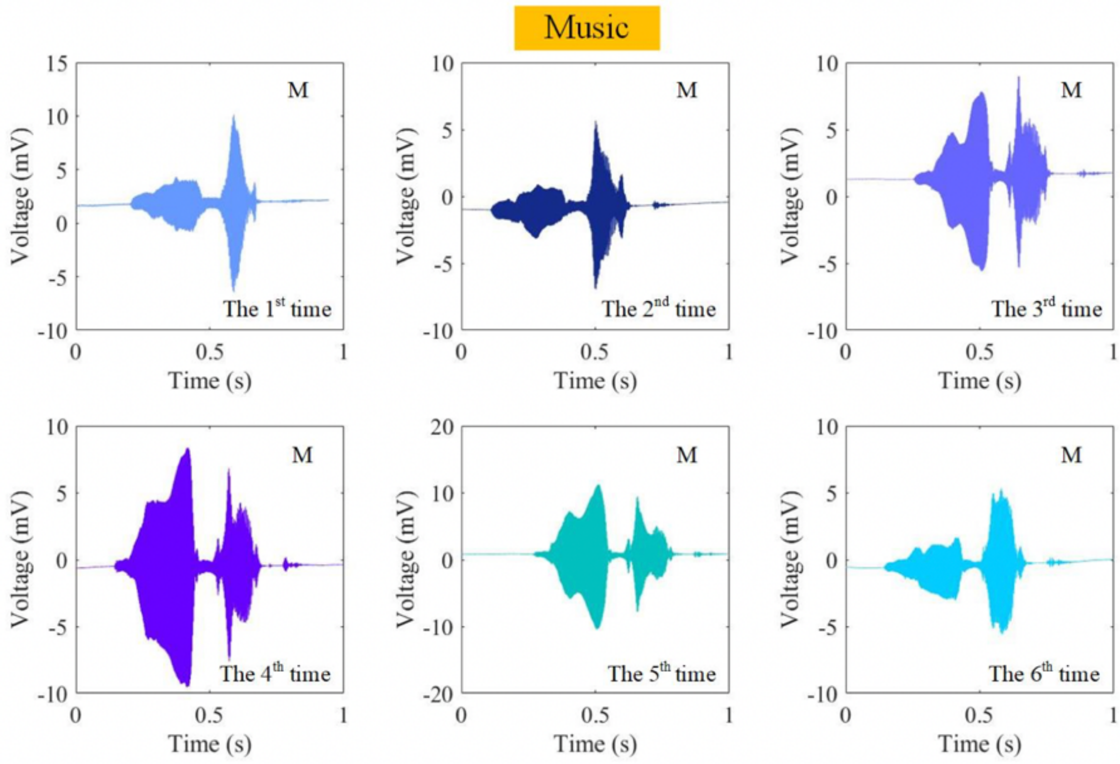

fig. S16. Temporal signals of the same word from the same person.

Comparison of the MBAS-recorded time traces for the same word ("music") pronounced by the same person.

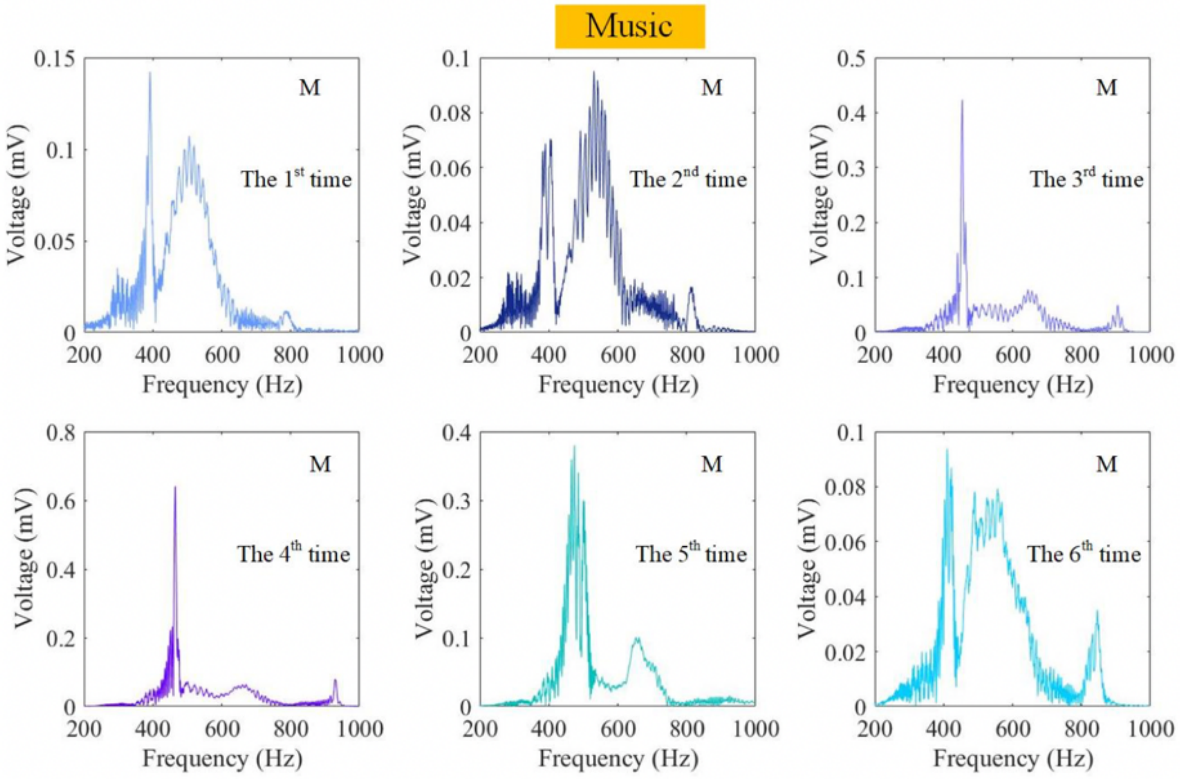

fig. S17. Frequency-domain signals of the same word from the same person.

Comparison of the MBAS-recorded frequency responses for the same word (“music”) pronounced by the same person.

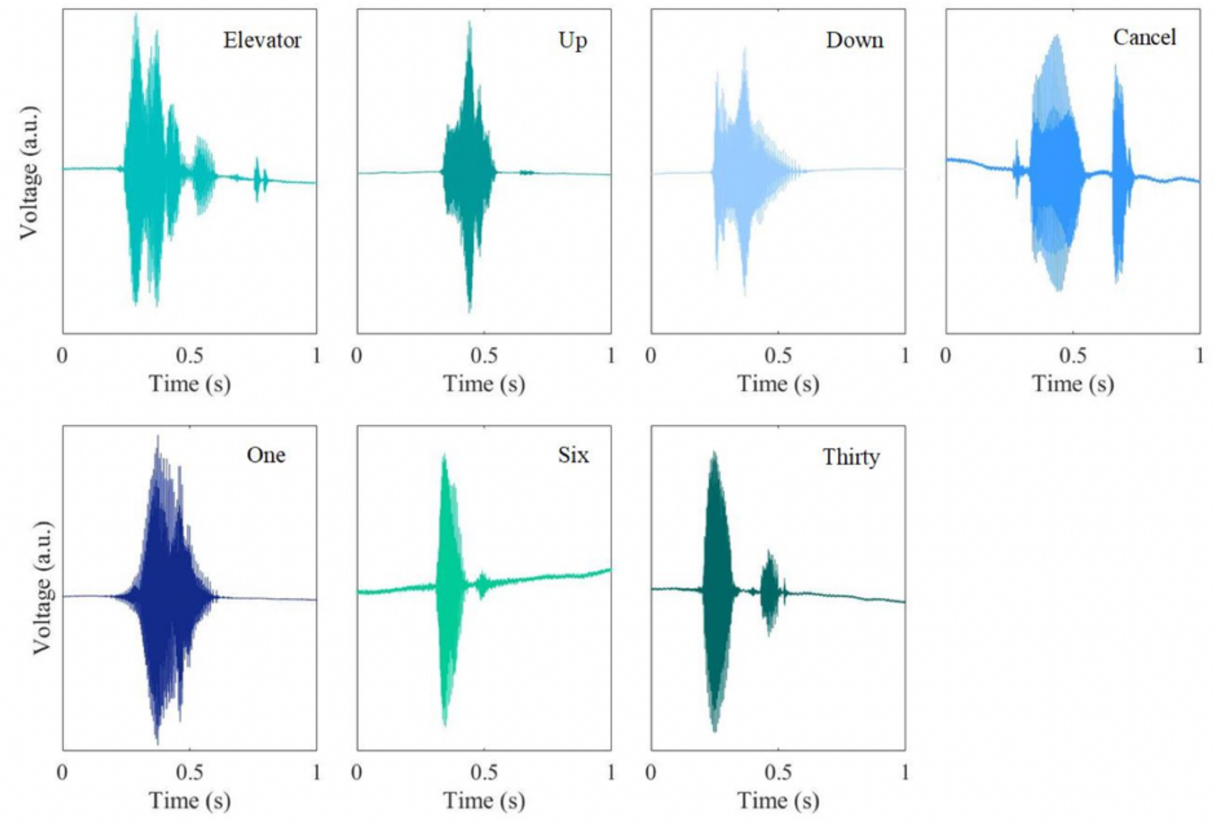

fig. S18. Temporal signals of different instructions.
MBAS-recorded time traces of seven elevator instructions pronounced by the same person.

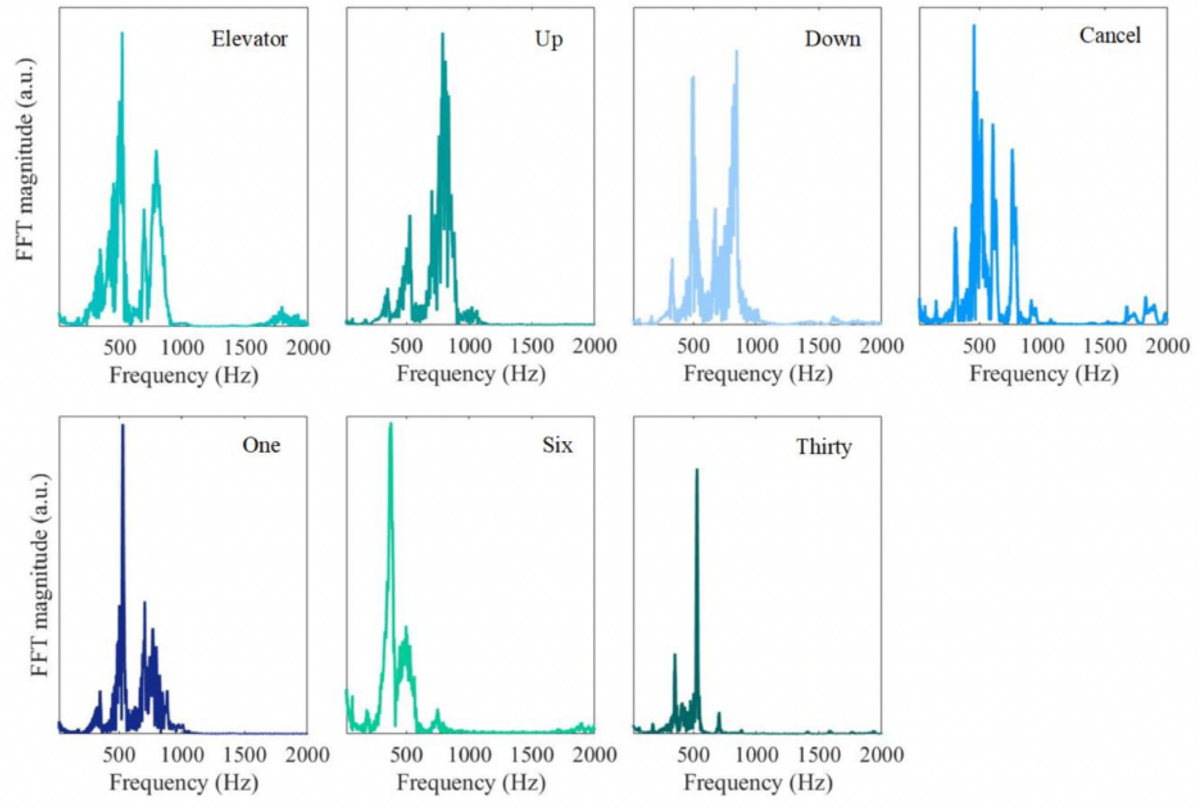

fig. S19. Frequency-domain signals of different instructions.

MBAS-recorded frequency responses for seven elevator instructions pronounced by the same person.

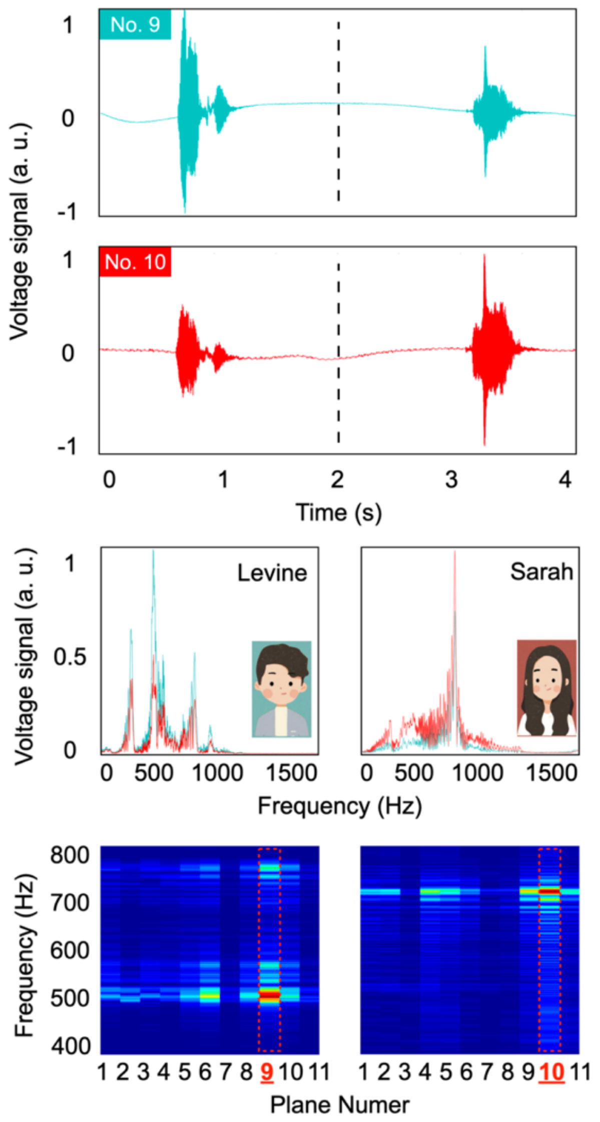

fig. S20.Temporal signals, FFT spectra, and normalized plane-frequency maps for separate talking and neighboring angles.

Levine spoke first at the position of plane No.9, which was shown as the signal of the first 2s in the time domain. At this time, the adjacent plane No.10 also had a small response. After that, Sarah repeated at the position of plane No.10, and the voltage signal was within 2 ~ 4s. Then, the source location and identity information could be clearly distinguished by FFT (Levine, 500 Hz; Sarah 700 Hz) and normalized plane frequency signal maps (Red in Plane No.9 and No.10).

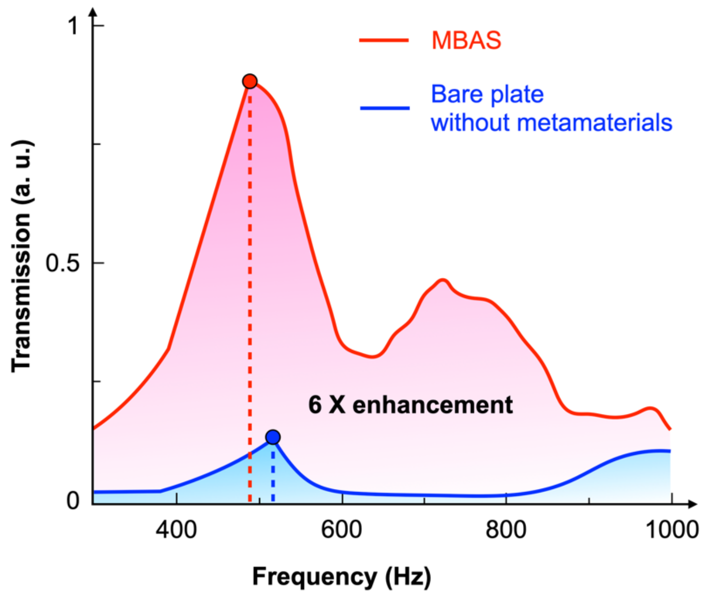

fig. S21. Signal enhancement via a metamaterial defect cavity compared to a bare transducer without metamaterials.

To eliminate the influence of sound pressure instability, the transmission spectra was characterized by the open-circuit voltage of unit sound pressure. It clearly shows that the peak response of MBAS is about 6 times higher than the peak response of the bare plate. Such an enhancement leads to the outstanding sensitivity achieved in this work.

Table S1. Material parameters of the planar acoustic metamaterial plates.

Parameters	Value
Silicone Rubber	
Density	1074 kg/m^3
Young Modulus	0.11 MPa
Poisson Ratio	0.49
Aluminum	
Density	2750 kg/m^3
Young Modulus	70 GPa
Poisson Ratio	0.33
Stainless Steel	
Density	7819 kg/m^3
Young Modulus	193 GPa
Poisson Ratio	0.25
PZT-5H	
Density	7500 kg/m^3
Compliance s_{11}	7.69×10^{-11} m^2/N
Compliance s_{12}	-4.78×10^{-12} m^2/N
Piezoelectric Constant d_{31}	-1.86×10^{-10} C/N
Piezoelectric Constant d_{33}	6.70×10^{-10} C/N
Relative Permittivity	3200

Table S2. Key dimensions of the acoustic metasphere structure.

Acoustic Metasphere	Value
Regular dodecahedron	
Volume	2.01×10^{-3} m^3
Surface Area	8.45×10^{-2} m^2
Radius of a circumscribed sphere	93.8 mm
Midradius	83.8 mm
Radius of an inscribed sphere	71.0 mm
Pentagon Plate	
Side Length	64 mm
Thickness	0.2 mm
Stainless Steel Columns	
Radius	4 mm
Height	5 mm
Silicone Rubber Columns	
Radius	4 mm
Height	5 mm
PZT-5H	
Radius	10 mm
Height	0.2 mm

Table S3. Comparison between metamaterial-based acoustic sensing systems.

Acoustic sensor systems integrated with metamaterial structures

Sensing Mechanism	System architecture	Design Novelty	Sensitivity (dBV)	Signal-to-Noise Ratio (dB)	Frequency (Hz)	Reference
**Wave compression**	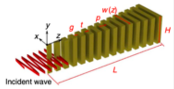	Sound pressure amplification	Used a commercial microphone	20 dB enhancement	> 7kHz	23
**Compressive sensing**	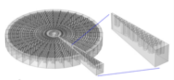	2D sound sources separation	Used a commercial microphone	Used a commercial microphone	3k ~ 5k Hz	25
**Cavity resonance**	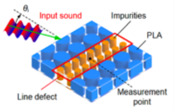	2D directional acoustic sensing	Used a commercial microphone	20 dB enhancement	2.6k ~ 3.7k Hz	48
**Compressive sensing**	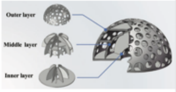	3D sound sources localization	Used a commercial microphone	Used a commercial microphone	100 ~ 5k Hz	26
**Randomized resonance**	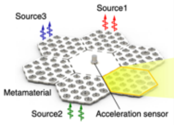	2D vibration localization	Used a commercial acceleration sensor	Unknown	304.0 ~ 493.7 Hz	27
**Helmholtz coupled resonator**	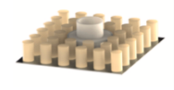	Acoustic energy harvesting	-39.26	Unknown	2k ~ 4k Hz	22
**Wave compression**	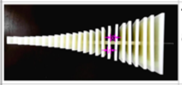	Tunable sound pressure amplification	Used a commercial microphone	9.47 dB enhancement	1.4k ~ 5.5k Hz	47
**Förster resonance**	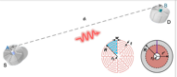	Passive remote-whispering	Used a commercial microphone	20.7 dB enhancement	525.5 ~ 600.5 Hz	24
**Wave confining and beamforming**	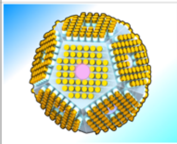	Omnidirectional pressure amplification and sound tracking	-26.30	21.75 dB enhancement (absolute value 71.65 dB)	200 ~ 1k Hz	This work

Table S4. Learning-based voice recognition under intense background noise in a factory.

Signal in different channels	Learning-based recognition results	Actual scenes	Background Noise
Plane No. 8	"WARNING"	"WARNING" (No. 8)	(1) Environmental noise;<br>(2) Air compressor noise;<br>(3) Alarm.
Plane No. 4	"HELP"	"WARNING" (No. 8) + "HELP" (No. 4)	
Plane No. 6	"HELP"	"WARNING" (No. 8) + "HELP" (No. 6)	
Plane No. 9	"HELP"	"WARNING" (No. 8) + "HELP" (No. 9)	

Movie S1.
Finite element simulation of the planar acoustic metamaterial plate.

Movie S2.
MBAS-based voice recognition system for authentication.

Movie S3.
MBAS-based high-quality recording system for Sonnet recitation.

Movie S4.
MBAS-based high-quality recording for classical music.

Movie S5.
MBAS-based fast and accurate recognition system for classical music.

Movie S6.
MBAS-based speech recording, recognition and tracking systems.
